# Machine learning to identify pairwise interactions between specific IgE antibodies and their association with asthma: A cross-sectional analysis within a population-based birth cohort

**DOI:** 10.1371/journal.pmed.1002691

**Published:** 2018-11-13

**Authors:** Sara Fontanella, Clément Frainay, Clare S. Murray, Angela Simpson, Adnan Custovic

**Affiliations:** 1 Section of Paediatrics, Department of Medicine, Imperial College London, London, United Kingdom; 2 Department of Epidemiology and Biostatistics, School of Public Health, Faculty of Medicine, Imperial College London, London, United Kingdom; 3 INRA, UMR1331, Toxalim, Research Centre in Food Toxicology, Toulouse, France; 4 Division of Infection, Immunity and Respiratory Medicine, Faculty of Biology, Medicine and Health, Manchester Academic Health Sciences Centre, University of Manchester and University Hospital of South Manchester NHS Foundation Trust, Manchester, United Kingdom; University of Virginia, UNITED STATES

## Abstract

**Background:**

The relationship between allergic sensitisation and asthma is complex; the data about the strength of this association are conflicting. We propose that the discrepancies arise in part because allergic sensitisation may not be a single entity (as considered conventionally) but a collection of several different classes of sensitisation. We hypothesise that pairings between immunoglobulin E (IgE) antibodies to individual allergenic molecules (components), rather than IgE responses to ‘informative’ molecules, are associated with increased risk of asthma.

**Methods and findings:**

In a cross-sectional analysis among 461 children aged 11 years participating in a population-based birth cohort, we measured serum-specific IgE responses to 112 allergen components using a multiplex array (ImmunoCAP Immuno‑Solid phase Allergy Chip [ISAC]). We characterised sensitivity to 44 active components (specific immunoglobulin E [sIgE] > 0.30 units in at least 5% of children) among the 213 (46.2%) participants sensitised to at least one of these 44 components. We adopted several machine learning methodologies that offer a powerful framework to investigate the highly complex sIgE–asthma relationship. Firstly, we applied network analysis and hierarchical clustering (HC) to explore the connectivity structure of component-specific IgEs and identify clusters of component-specific sensitisation (‘component clusters’). Of the 44 components included in the model, 33 grouped in seven clusters (C.sIgE-1–7), and the remaining 11 formed singleton clusters. Cluster membership mapped closely to the structural homology of proteins and/or their biological source. Components in the pathogenesis-related (PR)-10 proteins cluster (C.sIgE-5) were central to the network and mediated connections between components from grass (C.sIgE-4), trees (C.sIgE-6), and profilin clusters (C.sIgE-7) with those in mite (C.sIgE-1), lipocalins (C.sIgE-3), and peanut clusters (C.sIgE-2). We then used HC to identify four common ‘sensitisation clusters’ among study participants: (1) multiple sensitisation (sIgE to multiple components across all seven component clusters and singleton components), (2) predominantly dust mite sensitisation (IgE responses mainly to components from C.sIgE-1), (3) predominantly grass and tree sensitisation (sIgE to multiple components across C.sIgE-4–7), and (4) lower-grade sensitisation. We used a bipartite network to explore the relationship between component clusters, sensitisation clusters, and asthma, and the joint density-based nonparametric differential interaction network analysis and classification (JDINAC) to test whether pairwise interactions of component-specific IgEs are associated with asthma. JDINAC with pairwise interactions provided a good balance between sensitivity (0.84) and specificity (0.87), and outperformed penalised logistic regression with individual sIgE components in predicting asthma, with an area under the curve (AUC) of 0.94, compared with 0.73. We then inferred the differential network of pairwise component-specific IgE interactions, which demonstrated that 18 pairs of components predicted asthma. These findings were confirmed in an independent sample of children aged 8 years who participated in the same birth cohort but did not have component-resolved diagnostics (CRD) data at age 11 years. The main limitation of our study was the exclusion of potentially important allergens caused by both the ISAC chip resolution as well as the filtering step. Clustering and the network analyses might have provided different solutions if additional components had been available.

**Conclusions:**

Interactions between pairs of sIgE components are associated with increased risk of asthma and may provide the basis for designing diagnostic tools for asthma.

## Abstract 2

Adnan Custovic and colleagues demonstrate that combinations of allergen sensitivities in children can be used to predict concurrent asthma diagnosis.

## Author summary

**Why was this study done?:**

- The relationship between allergic sensitisation and asthma is complex.
- Asthma prediction models based on the IgE responses to the whole allergen extracts exhibit relatively poor performance.
- This study examines the relationship between IgE responses to multiple allergen components in component-resolved diagnostics (CRD) and their associations with asthma.

**What did the researchers do and find?:**

- Serum-specific IgE responses to 112 allergen components were measured using a multiplex array among children in a population-based birth cohort.
- Researchers applied network analysis and hierarchical clustering (HC) to explore the connectivity structure of component-specific IgEs and identified seven clusters of component-specific sensitisation. Cluster membership mapped closely to the structural homology of proteins and/or their biological source.
- HC identified four ‘sensitisation clusters’ among study participants.
- The relationship between component clusters, sensitisation clusters, and asthma was explored using a bipartite network.
- The differential network of pairwise component-specific IgE interactions was inferred, which demonstrated that interactions among 18 pairs of allergen components predicted asthma with a good balance between sensitivity and specificity. For example, children with IgE antibodies to different allergenic proteins from both dog and cat, or horse and house dust mite, are at higher risk of developing asthma.

**What do these findings mean?:**

- IgE responses to multiple allergenic proteins are functionally coordinated and co-regulated.
- Pairwise interactions within this complex network predict clinical phenotypes. Interactions between pairs of sIgE components are associated with increased risk of asthma and provide the basis for designing diagnostic tools for asthma.

## Introduction

Asthma is the most common noncommunicable disease in childhood. Over recent decades, a large body of evidence has demonstrated a close relationship between specific immunoglobulin E (sIgE) antibody responses and asthma [1, 2], but the data about the strength of this association are conflicting [2, 3]. Furthermore, in a clinical situation, confirmation of allergic sensitisation using standard diagnostic tests (skin prick tests [SPTs] and/or measurement of sIgE) does not necessarily indicate that patient's symptoms are caused by an allergic reaction [1]. We have previously proposed that these inconsistencies are in part consequent to ‘allergic sensitisation’ not being a single entity (as considered conventionally) but an umbrella term for a collection of several different classes of sensitisation that differ in their association with asthma and other allergic diseases. To test this, in a previous study we applied a machine learning approach with Bayesian inference to a comprehensive set of skin tests and sIgE data to whole allergen extracts collected from infancy to school age in a population-based birth cohort [4]. Children clustered into four distinct sensitisation classes characterised by different patterns of responses to specific allergens and the time of onset of sensitisation [4]. The risk of asthma was increased almost 30-fold amongst children belonging to one of these classes (assigned as ‘Multiple early sensitisation’, comprising less than one third of children diagnosed as sensitised using conventional definitions). We have replicated these findings in another birth cohort [5] and have shown that diminished lung function in adolescence and early adulthood is associated with ‘Multiple early’, but not other sensitisation classes [6, 7].

In food allergy, there is increasing evidence that sensitisation to some, but not all, allergenic proteins in allergen extracts is important for making a distinction between true allergy and asymptomatic sensitisation [8]. For example, we have shown that immunoglobulin E (IgE) response to peanut protein *Ara h 2* is much more predictive of true peanut allergy than standard tests using whole allergen extract [9, 10]. Measuring sensitisation to these individual molecules (referred to as allergen components) using component-resolved diagnostics (CRD) may be more informative than standard tests in respiratory allergy, as well. The developments in molecular diagnostics have led to products such as the multiplex Immuno Solid-phase Allergen Chip (ImmunoCAP ISAC), in which sIgE to more than 100 allergen components can be measured simultaneously [11]. Using a machine learning approach, we have shown that patterns of component-specific IgE responses in this multiplex assay have reasonable discrimination ability for asthma and rhino-conjunctivitis [12]. In a further study using latent variable modelling, we identified several cross-sectional clusters of IgE responses in school age children, and each of these clusters was associated with different clinical symptoms [13]. Our subsequent study using nested latent class probabilistic modelling has indicated that longitudinal trajectories of sensitisation to several grass and house dust mite (HDM) allergens during childhood had different associations with clinical outcomes [14].

Based on these findings, we propose (1) that the impact of allergic sensitisation on asthma is a complex phenomenon that cannot be captured by considering individual allergen sIgE responses separately, or in isolation; and (2) that sIgE responses to multiple allergenic proteins are functionally coordinated and co-regulated, and this complex network of interactions foreshadows asthma development. Specifically, we hypothesise that interaction patterns between component-specific IgE antibodies rather than individual IgE responses to ‘informative’ components are associated with risk of asthma. To address our hypothesis, we measured sIgEs to 112 allergen components using a commercially available multiplex array among participants in a population-based birth cohort, and we used unsupervised machine learning techniques to explore how component-specific IgEs interact with each other and to identify common sensitisation profiles among children. We then used a supervised machine learning approach to explore interactions of component-specific IgEs in relation to asthma.

## Materials and methods

### Study design, setting, and participants

The Manchester Asthma and Allergy Study is a population-based birth cohort [15]. Participating families were recruited from the maternity catchment area of Wythenshawe and Stepping Hill Hospitals in South Manchester and Cheshire, United Kingdom [15]. All pregnant women were screened for eligibility at antenatal visits (8th–10th week of pregnancy) between 1 October 1995 and 1 July 1997. Of the 1,499 women and their partners who met the inclusion criteria, 288 declined to take part in the study, and 27 were lost to follow-up between recruitment and childbirth. The study was approved by the Research Ethics Committee and parents gave written informed consent.

### Data sources/Measurement and definition of outcomes

Children attended review clinics at ages 1, 3, 5, 8, 11, and 16 years. Validated questionnaires were interviewer administered to determine parentally reported history of wheeze, eczema, and rhinitis, and treatments received. SPT was used to ascertain atopic sensitisation to common inhalant and food allergens, and lung function measurements were obtained using spirometry at all visits from age 5 years. A blood sample was collected in children who gave assent for venepuncture [16]. Primary care medical records were examined and data including wheeze episodes, prescriptions of asthma medications and oral corticosteroid, and hospitalisations were extracted.

In this study, we performed a cross-sectional analysis using data collected at age 11 years.

‘Current wheeze’ was defined as a positive answer to the question, ‘Has your child had wheezing or whistling in the chest in the last 12 months?’ [17] ‘Current asthma’ was defined as a positive answer to two out of three of: ‘Has the doctor ever told you that your child had asthma?’; ‘Has your child had wheezing or whistling in the chest in the last 12 months?’; and ‘Has your child had asthma treatment in the last 12 months?’ [18]. Further details of follow-up and definitions of clinical outcomes are presented in the supplementary appendix (S1 Appendix).

### CRD

We measured sIgE to 112 allergenic molecules using ImmunoCAP ISAC (Thermo Fisher Scientific-Phadia AB, Uppsala, Sweden) at the follow-up at age 11 years. The level of component-specific IgE antibodies was reported in ISAC Standardised Units (ISU). To ascertain co-occurring sensitisations among participants, we dichotomised IgE data according to the manufacturer's guidelines, using a binary threshold (positive>0.30 ISU). To evaluate the differential connectivity structure of component-specific IgEs, we used continuous raw values.

### Statistical learning

In this cross-sectional analysis, we included all children with available CDR data. We analysed data for components with sIgE>0.30 ISU in at least 5% of children (active components) and among participants with at least one active component sIgE>0.30 ISU (filtering) [19]. A flowchart describing the analysis steps involved in this study is presented in S1 Fig.

### Statistical grouping of allergen components and their connectivity structure: Component clusters

We investigated patterns of sIgE co-expression using hierarchical clustering (HC), which transforms a distance matrix into a nested series of partitions that can be represented through a treelike graph (dendogram). By exploring this graph, one can obtain useful information on the hierarchy of the clusters and their similarities. At the lowest level of the hierarchy, each cluster contains a single observation. At the highest level, there is only one cluster containing all of the data. HC algorithms can follow an agglomerative or a divisive approach. Agglomerative strategies start at the bottom and at each level recursively merge a selected pair of clusters into a single cluster. This produces a grouping at the next higher level with one fewer cluster. The pair chosen for merging consist of the two groups with the smallest intergroup dissimilarity. Divisive methods start at the top and at each level recursively split one of the existing clusters at that level into two new clusters. The split is chosen to produce two new groups with the largest between-group dissimilarity. With both paradigms there are *N*−1 levels in the hierarchy [20]. In our analysis, we used the agglomerative procedure combined with the average linkage method, which defines the distance between two clusters as the average distance between each point in one cluster to every point in the other cluster.

Compared with partitional clustering, HC techniques do not require one to fix the number of clusters a priori, can find different levels of similarity between the sIgE components within the hierarchy of clusters, and, hence, can highlight different patterns of connectivity and biological properties.

Distances between sIgE components were expressed by means of the distance correlation matrix [21]. The advantage of using distance correlation is that it is capable of detecting nonlinear relationships. We then used network analysis to visualise the connectivity structure of sIgEs.

Final partitions can significantly differ according to the chosen clustering approach. Hence, to evaluate the robustness of our findings, we compared the retrieved clusters with partitions obtained through a divisive HC procedure and a partitional clustering technique using the Rand index [22].

### Patterns of sensitisation among study participants: Sensitisation clusters

To identify patterns of sensitisation among children, we used an HC approach combined with Ward's linkage [23] and the Jaccard distance between binary responses to sIgE profiles. At each iteration of the clustering algorithm, the Ward's method joins the clusters so that the total within-cluster variance is minimised. Ward's linkage is conservative, monotone, correctly infers the hidden structure within the data, and often outperforms the other approaches [24, 25]. We used *χ*^2^ and Kruskal–Wallis tests to evaluate the associations between the identified clusters and clinical outcomes.

### Differential sIgE co-expression patterns in asthma

We used a bipartite network to visually explore the relationship between component clusters, sensitisation clusters, and asthma. We investigated whether sIgE to individual components is associated with the risk of asthma using a penalised logistic regression model. To test the hypothesis that pairwise interactions of component-specific IgEs are associated with asthma, we used joint density-based nonparametric differential interaction network analysis and classification (JDINAC) [26]. We utilised this recently developed nonparametric model to identify differential interaction patterns of network activation of sIgEs that are most closely related to asthma, and to build a classification model using the network biomarkers. JDINAC has the advantage of capturing nonlinear relations between component-specific IgEs without the need for parametric assumption on their probability distribution.

The main assumption of the JDINAC model is that network-level difference between children who have asthma and children who do not have asthma arises from the collective effect of differential pairwise component IgE interactions. Here, the interactions are characterised by the conditional joint density of pairs of component-specific IgEs [26], estimated through a nonparametric kernel method. Formally, let **X**_*n*×*p*_ be the data matrix of *n* individuals and *p* sIgE allergens. Hence, *X*_*l*_, *l* = 1,…,*n*, represents the level of sIgEs in the *l*-th child. Let *Y*_*l*_ denote the binary variable defined as follows:

$$Y_{l}=\{\begin{array}{l} 0\;if\;l\;is\;non-asthmatic\\ 1\;if\;otherwise \end{array}$$

Let *P* denote the probability of having asthma, *P* = *Pr*(*Y*_*l*_ = 1), and *G*_*i*_ denote the *i*-th sIgE. Then, JDINAC logistic regression-based approach can be exploited to test the model:

$$logit(P)=\alpha_{0}+\sum_{i=1}^{p}\sum_{j>i}\beta_{ij}\ln\frac{f_{ij}^{1}(G_{i},G_{j})}{f_{ij}^{0}(G_{i},G_{j})},\;s.t.\;\sum_{i=1}^{p}\sum_{j>i}\beta_{ij}\leq c,\;c>0$$

where $f_{ij}^{1}(G_{i},G_{j})$ and $f_{ij}^{0}(G_{i},G_{j})$ denote the class conditional joint density of *G*_*i*_ and *G*_*j*_ for class 1 and class 0, respectively. The conditional joint densities $f_{ij}^{1}(G_{i},G_{j})$ indicate the strength of association between *G*_*i*_ and *G*_*j*_ in class 1, and parameters *β*_*ij*_ indicate differential dependency patterns between condition-specific groups [26]. The estimation procedure is based on a multiple splitting and prediction averaging procedure, which guarantees robust and accurate results. The data are split in two parts. On the first part, joint kernel density functions, ${\hat{f}}_{ij}^{1}$ and ${\hat{f}}_{ij}^{0}$, are estimated, while on the second part, *L*_1_ penalised logistic regression is fitted. The procedure is repeated for a predefined number of iterations (for estimation details and algorithm, see [26]).

To ensure robustness of the results, we ran both models with 10-fold cross validation in 50 independent repetitions. To reduce the effect of imbalanced data, we included class weight in both models. sIgE raw values were log-transformed (log(*x*+1)) prior to these analyses.

### Validation

To evaluate the robustness of our results and provide external narrow validation [27], we repeated the analysis among cohort participants who had ISAC CRD data at age 8 years, excluding the children whose data were used in the primary analysis at age 11 years. For children in the validation step, both CDR data and clinical outcomes were ascertained at age 8 years.

All statistical analyses were run in the programming language R [28]. Distance correlation was computed with the package *energy* [29]. JDINAC scripts were made available by the authors [26] at https://github.com/jijiadong/JDINAC. We used *igraph* package for network visualisations [30], *epitools* to estimate the odds ratio (OR) [31], *clValid* to compute internal validity measures for HC [32], and *caret* to infer the penalised logistic regression model [33].

## Results

### Participant flow and demographic data

Among 1,184 children born into the cohort, 822 attended clinical follow-up at age 11 years. CRD data were obtained for 461 (56.1%) children. Demographics of these 461 participants are presented in S1 Table; we have also previously reported that there were no significant differences in demographic characteristics or outcomes between cohort members with and without CRD [13]. Of 461 children with CRD, 221 (47.9%) had positive sIgE to at least one of the 112 allergen components [13], and 94 (20.4%) had current asthma. After filtering [19], 44/112 allergen components were active; 213 (46.2%) children had at least one of the active component IgEs >0.30 ISU, 73 (34.3%) of whom had asthma. The list of components that were inactive [19] and the proportion of children who had positive sIgE to these ‘rare’ components are presented in S2 Table.

**Fig 1:**
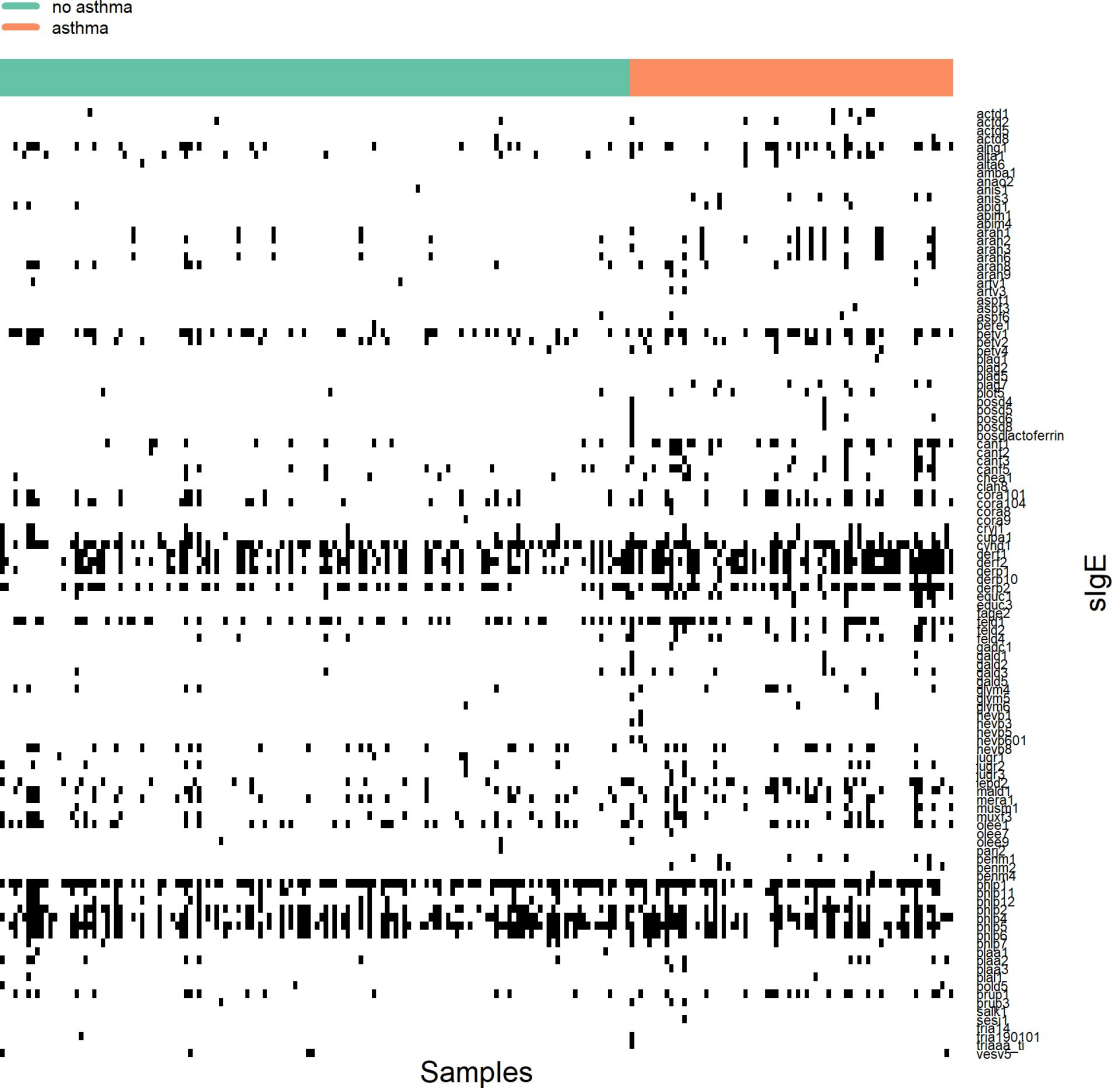
Patterns of sensitisations stratified by asthma status. Participants are represented in columns and sIgE components in rows. A black square indicates that a participant has a sIgE>0.30 to a particular allergen component. sIgE, specific immunoglobulin E.

There was a significant difference in the total number of positive component-specific IgEs between children who have asthma and children who do not have asthma, with children who have asthma responding to more allergens than children who do not have asthma (median 11 [IQR: 6–18] versus 6 [IQR: 3–10 ], *p*<0.001, S2 Fig). The responses to individual components stratified by disease status did not show considerable differences between sensitised children with and without asthma (Fig 1). However, we highlight an increase in the positive responses to some allergenic proteins among children who have asthma, particularly group 2 HDM components and furry animal lipocalins (S3 Table).

### Statistical grouping of allergen components (component clusters) and their connectivity structure

Of the 44 allergen components included in the model, 33 grouped in seven component clusters (C.sIgE-1–7), while the remaining 11 formed singleton clusters (Table 1). The number of clusters was determined by fixing the threshold for the dissimilarity measure (1−distance correlation) equal to 0.40, which ensured high similarity between the components.

**Table 1:** Component-specific IgE clusters membership.

Component clusters	sIgEs	Species	Biochemical name
*C.sIgE-1	*Der p 1*	*Dermatophagoides pteronyssinus* (European HDM)	Cysteine protease
	*Der p 2*	*D*. *pteronyssinus* (European HDM)	NPC2 family
	*Der f 1*	*D*. *farinae* (American HDM)	Cysteine protease
	*Der f 2*	*D*. *farinae* (American HDM)	NPC2 family
*C.sIgE-2	*Ara h 1*	*Arachis hypogaea* (Peanut, groundnut)	Cupin (Vicillin-type, 7S globulin)
	*Ara h 2*	*A*. *hypogaea* (Peanut, groundnut)	Conglutin (2S albumin)
	*Ara h 6*	*A*. *hypogaea* (Peanut, groundnut)	Conglutin (2S albumin)
*C.sIgE-3	*Fel d 4*	*Felis domesticus* (Cat)	Lipocalin
	*Equ c 1*	*Equus caballus* (Domestic horse)	Lipocalin
	*Can f 1*	*Canis familiaris* (Dog)	Lipocalin
	*Mus m 1*	*Mus musculus* (Mouse)	Lipocalin
*C.sIgE-4	*Phl p 1*	*Phleum pratense* (Timothy)	Beta-expansin
	*Phl p 2*	*P*. *pratense* (Timothy)	Grass group II/III
	*Phl p 4*	*P*. *pratense* (Timothy)	Berberine bridge enzyme
	*Phl p 5*	*P*. *pratense* (Timothy)	
	*Phl p 6*	*P*. *pratense* (Timothy)	
	*Cyn d 1*	*Cynodon dactylon* (Bermuda grass)	Beta-expansin
*C.sIgE-5	*Gly m 4*	*Glycine max* (Soybean)	PR-10
	*Mal d 1*	*Malus domestica* (Apple)	PR-10
	*Aln g 1*	*Alnus glutinosa* (Alder)	PR-10
	*Bet v 1*	*Betula verrucosa* (*B*. *pendula*) (White birch)	PR-10
	*Pru p 1*	*Prunus persica* (Peach)	PR-10,
	*Cor a 1*.*04*	*Corylus avellana* (Hazelnut)	2S albumin
	*Ara h 8*	*A*. *hypogaea* (Peanut, groundnut)	PR-10,
	*Cor a 1*.*01*	*C*. *avellana* (Hazelnut)	PR-10,
*C.sIgE-6	*Cup a 1*	*Cupressus arizonica* (Cypress)	Pectate lyase
	*Jug r 2*	*Juglans regia* (English walnut)	Vicilin seed storage protein
	*Pla a 2*	*Platanus acerifolia* (London plane tree)	Polygalacturonase
	*Cry j 1*	*Cryptomeria japonica* (Sugi)	Pectate lyase
*C.sIgE-7	*Mer a 1*	*Mercurialis annua* (Annual mercury)	Profilin
	*Bet v 2*	*B*. *verrucosa* (*B*. *pendula*) (European white birch)	Profilin
	*Hev b 8*	*Hevea brasiliensis* (Para rubber tree [latex])	Profilin
	*Phl p 12*	*P*. *pratense* (Timothy)	Profilin
*Singletons	*Der p 10*	*D*. *pteronyssinus* (European HDM)	Tropomyosin
	*Lep d 2*	*Lepidoglyphus destructor* (Storage mite)	NPC2 family
	*Fel d 1*	*F*. *domesticus* (Cat)	Uteroglobin (chain 1)
	*Blo t 5*	*Blomia tropicalis* (Storage mite)	
	*Gal d 3*	*Gallus domesticus* (Chicken)	Ovotransferrin
	*Phl p 11*	*P*. *pratense* (Timothy)	Ole e 1–related protein
	*Mux f 3*	Bromelain	
	*Che a 1*	*Chenopodium album* (Lambsquarters)	Ole e 1 homologue
	*Ole e 1*	*Olea europaea* (Olive)	Common olive group 1
	*Can f 5*	*C*. *familiaris* (Dog)	Arginine esterase, prostatic kallikrein

**Abbreviations:** HDM, house dust mite; IgE, immunoglobulin E; PR, pathogenesis-related.

We compared the adopted model with the divisive HC clustering DIANA (Divise Analysis) [34], and the partition around medoids (PAM) [34] algorithm. The Rand index, 0.99 for DIANA and 0.98 for PAM, suggested that the obtained groups were stable and robust. Internal validity indices also showed that cluster membership was very stable (S3 Fig).

C.sIgE-1 was composed exclusively of HDM components (Group 1 and 2 HDM allergens); C.sIgE-2 of peanut components associated with true peanut allergy (2S albumins and 7S globulin) [9]; C.sIgE-3 of lipocalins from cat, dog, horse, and mouse; C.sIgE-4 of grass components; C.sIgE-5 of PR-10 proteins from various sources; C.sIgE-6 of tree allergens; and C.sIgE-7 of profilins. The HC highlighted the structural relationships of the allergen components within protein families.

**Fig 2:**
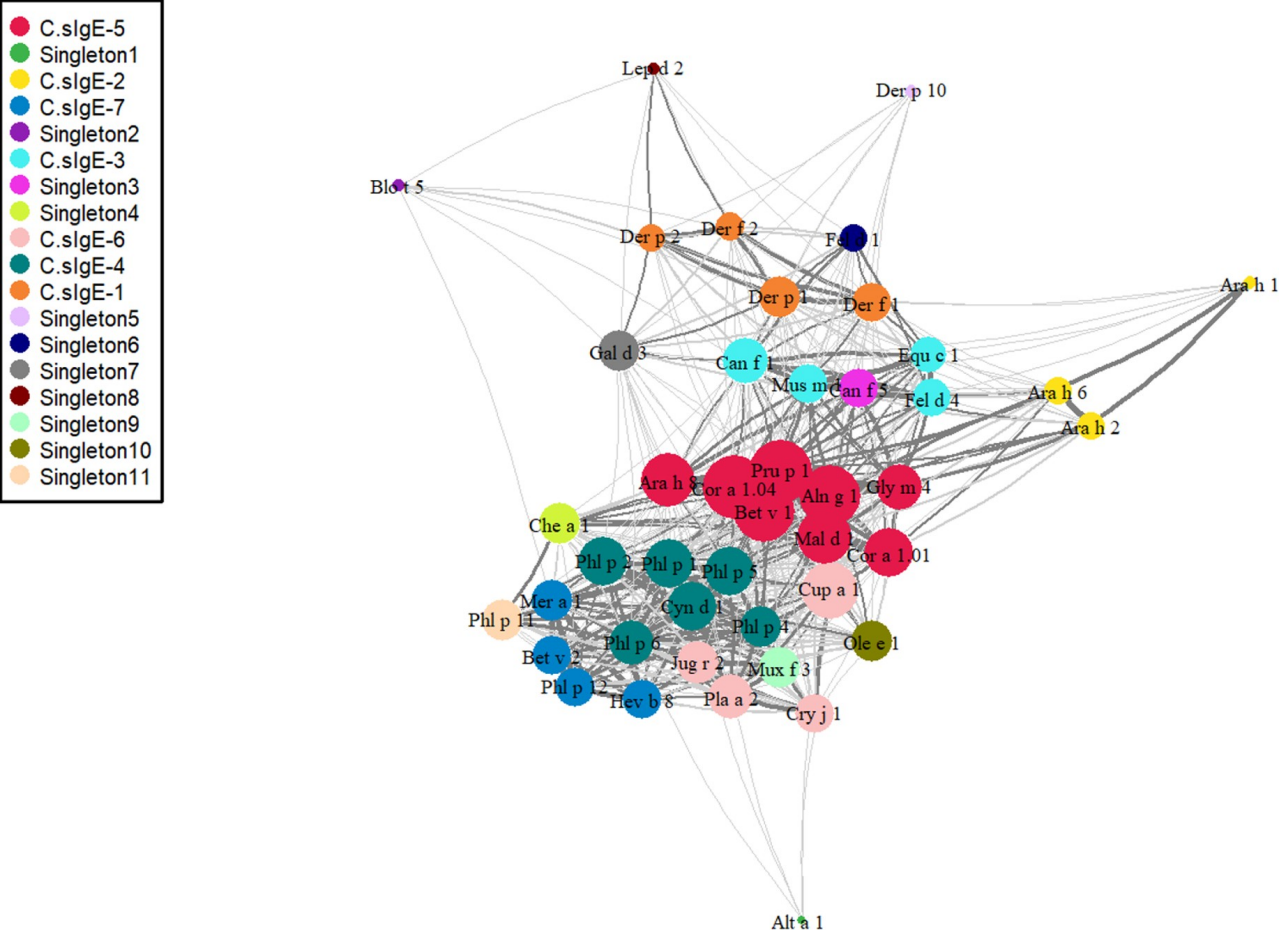
Component-specific IgE network and hierarchical cluster reveal connectivity structure in sIgE. The network consists of a set of nodes, joined in pairs by lines or edges. Colours represent cluster memberships and node diameter is proportional to the scaled connectivity of each sIgE, while edge colour and width represent the strength of connection between pairs of sIgE components. IgE, immunoglobulin E; sIgE, specific immunoglobulin E.

The co-expression network in Fig 2 shows the interactions and underlying connectivity structure of component-specific IgEs. The connectivity expresses how sIgE components are correlated and co-regulated with each other. Components belonging to the PR-10 (C.sIgE-5) cluster were central to the network, showing higher connectivity than other components; components in this cluster seem to mediate connections between components from grass (C.sIgE-4), tree (C.sIgE-6), and profilin (C.sIgE-7) clusters with components in HDM (C.sIgE-1), lipocalins (C.sIgE-3), and peanut clusters (C.sIgE-2). *Alt a 1* and *Blo t 5* were weakly connected to other component-specific IgEs. Components in the HDM cluster showed high intraclass connectivity.

### Characteristics of sensitisation profiles (sensitisation clusters) among study participants

The structure of sensitisation profiles among study participants was inferred in a completely unsupervised manner, with the optimal solution suggesting four sensitisation clusters (based on the Calinski-Harabasz criterion [35]). Cluster membership was stable (S4 Fig). In the model comparisons, the Rand index showed moderate agreement with the partition obtained with DIANA (0.53) and good agreement with the partition obtained with PAM (0.79).

**Fig 3:**
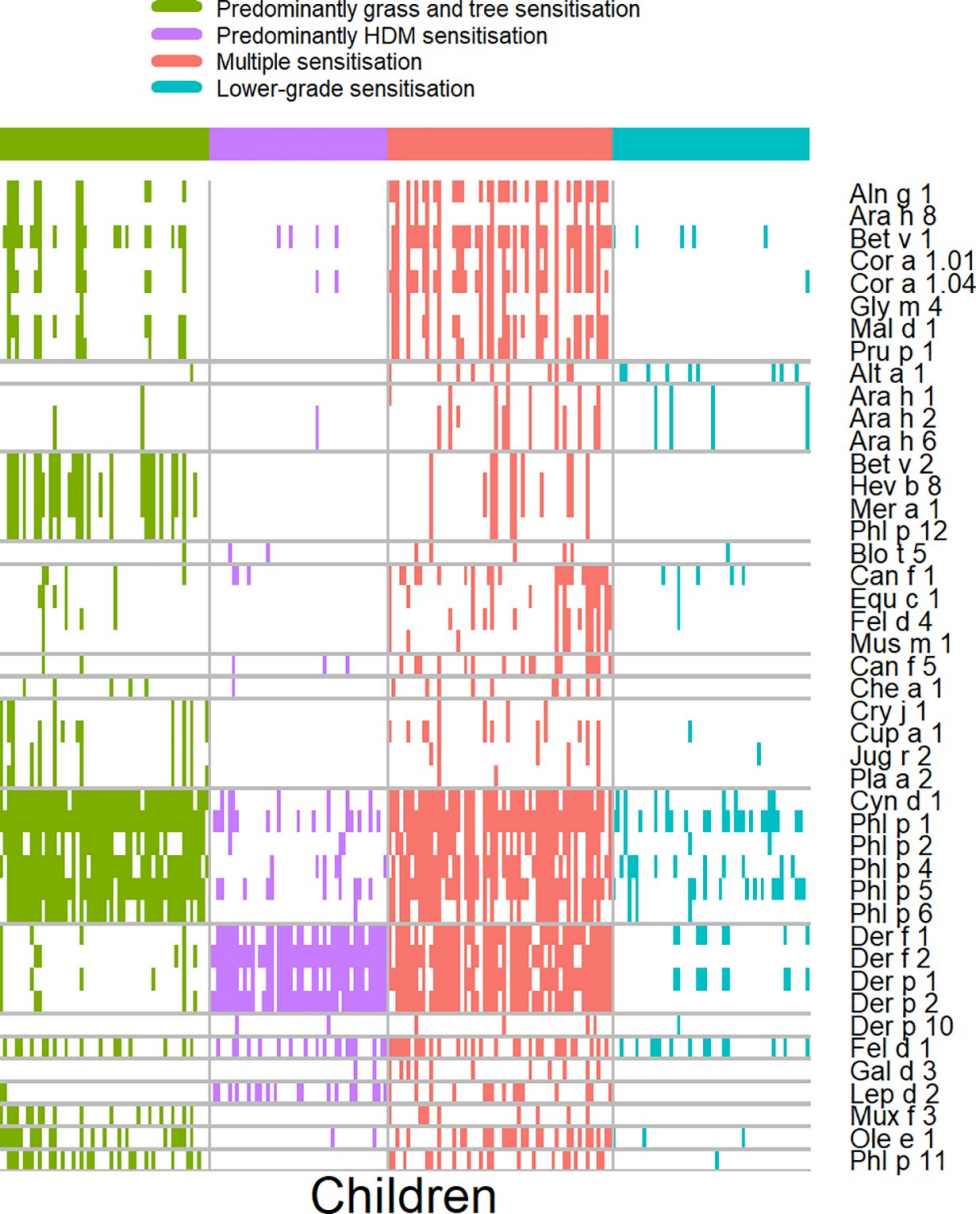
Patterns of IgE responses to allergen components for individual participants. Rows represent sIgEs, while columns indicate children. Colours represent sensitisation clusters’ membership. Squares are coloured if and only if a child has a positive response, <0.30 to a particular sIgE. IgE, immunoglobulin E; sIgE, specific immunoglobulin E.

After visual inspection of the patterns (Fig 3), we labelled these four sensitisation profiles as (1) Multiple sensitisation, with positive sIgE to multiple components across all seven component clusters (C.sIgE-1–7) and singleton components; (2) Predominantly HDM sensitisation, with IgE responses mainly to components from C.sIgE-1; (3) Predominantly grass and tree sensitisation, with positive sIgE to multiple components across C.sIgE-4–7; and (4) Lower-grade sensitisation.

Association with clinical outcomes (asthma, rhinitis, and atopic dermatitis [AD]) differed for different sensitisation profiles (S4 Table, S5 Table). Children in the HDM cluster were more likely to have asthma (OR: 4.44; 95% CI: 1.72–11.46; *p* = 0.002) and wheeze (OR: 7.31; 95% CI: 2.74–19.48; *p* < 0.001), but not rhinitis or AD, while those in the grasses/trees cluster were more likely to have rhinitis (OR: 6.62; 95% CI: 2.84–15.40; *p* < 0.001). Membership of the Multiple sensitisation cluster was associated with the highest risk of asthma (OR: 4.97; 95% CI: 1.99–12.34; *p* < 0.001) and a high risk of wheeze (OR: 4.41; 95% CI: 1.70–11.41; *p* < 0.001) and rhinitis (OR: 6.18; 95% CI: 2.71–14.12; *p* < 0.001) (S5 Table). No significant associations were found with lung function measurements (S6 Table).

### Differential sIgE co-expression patterns in the prediction of asthma

**Fig 4:**
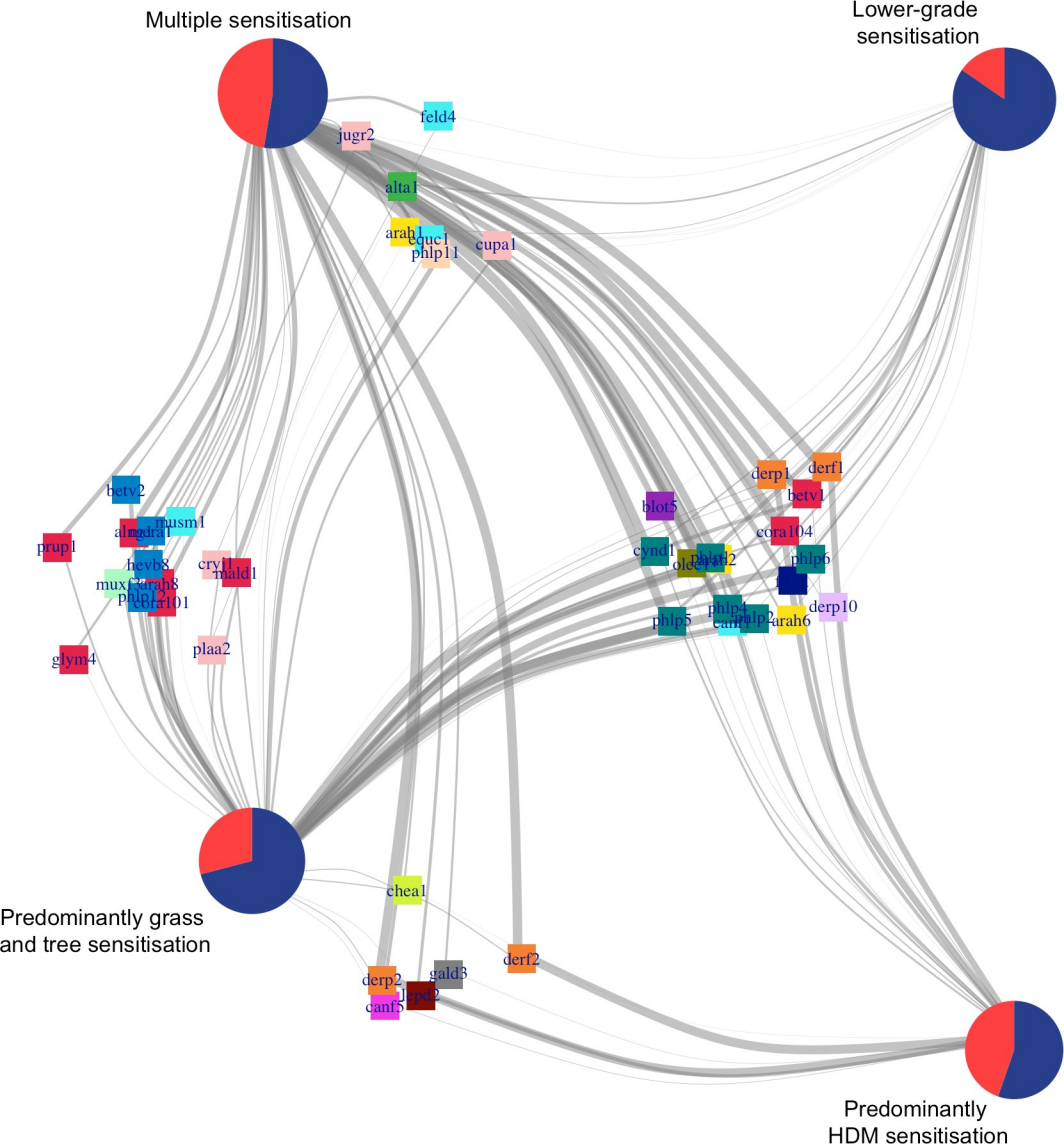
Bipartite network to uncover the relationship between sensitisation clusters and asthma, and the connectivity with component-specific IgEs and component clusters. In the bipartite network, nodes represent one or more types of entities, and edges between the nodes represent a specific relationship between the entities. Here, pie charts represent individuals aggregated according to sensitisation cluster membership and asthma status. Red indicates children with asthma, while blue indicates no asthma. Squares represent sIgE allergens and colours represent cluster membership. Edges show whether a subject has a positive response to a particular c-sIgE. MDS layout was used to infer the network. HDM, house dust mite; IgE, immunoglobulin E; MDS, multidimensional scaling; sIgE, specific immunoglobulin E.

Fig 4 summarises the relationship between sensitisation clusters and asthma, and the connectivity with component-specific IgEs and component clusters. Although a significantly higher proportion of children with asthma was found in the Multiple sensitisation and HDM clusters, the majority of children in each of the sensitisation clusters did not have asthma. All clusters shared similar connection to some component clusters (C.sIgE-3 and C.sIgE-4), but we observed distinct patterns of connectivity between the cluster with a higher proportion of children with asthma compared with those with a higher proportion of children who did not have asthma. Specifically, only children in Multiple sensitisation and Predominantly HDM clusters were strongly connected to the allergens in C.sIgE-1, while children in Predominantly grasses/trees and Lower-grade sensitisation clusters were distinctively connected to C-sIgE-2.

S5 Fig shows examples of bipartite subnetworks of a subset of component clusters. Panel A shows the connectivity between a set of informative components in the lipocalin cluster (C.sIgE-3) with *Fel d 1*. The analysis has shown that children with connection to only one sIgE were not at higher risk of asthma, but those who were connected to two or more components were at increased risk of having asthma. Similar behaviours are observed for all the other networks, apart from interactions involving the grass IgE cluster (C.sIgE-4).

To investigate whether individual components sIgE or pairwise interactions of component-specific IgEs are stronger associates of asthma, we compared the performances of penalised logistic regression and JDINAC in classifying asthma (Table 2). In the multivariate logistic regression model, we include all the 44 individual components as predictors. To improve comparability between the two models, a penalty on the *L*1-norm was included in the logistic model.

**Table 2:** Evaluation and comparison of prediction performances of logistic regression based on individual components and JDINAC based on pairwise interactions of sIgE allergens.

Performance metrics	Age 11		Age 8	
	Penalised logistic regression	JDINAC	Penalised logistic regression	JDINAC
	individual components	pairwise interactions	individual components	pairwise interactions
**AUC**	0.73	0.94	0.62	0.97
**Accuracy**	0.67	0.86	0.71	0.92
**Sensitivity**	0.60	0.84	0.46	0.79
**Specificity**	0.70	0.87	0.85	0.98
**Precision**	0.51	0.78	0.61	0.97
**F measure**	0.55	0.81	0.52	0.86

Abbreviations: AUC, area under the curve; JDINAC, joint density-based nonparametric differential interaction network analysis and classification; sIgE, specific immunoglobulin E.

**Fig 5:**
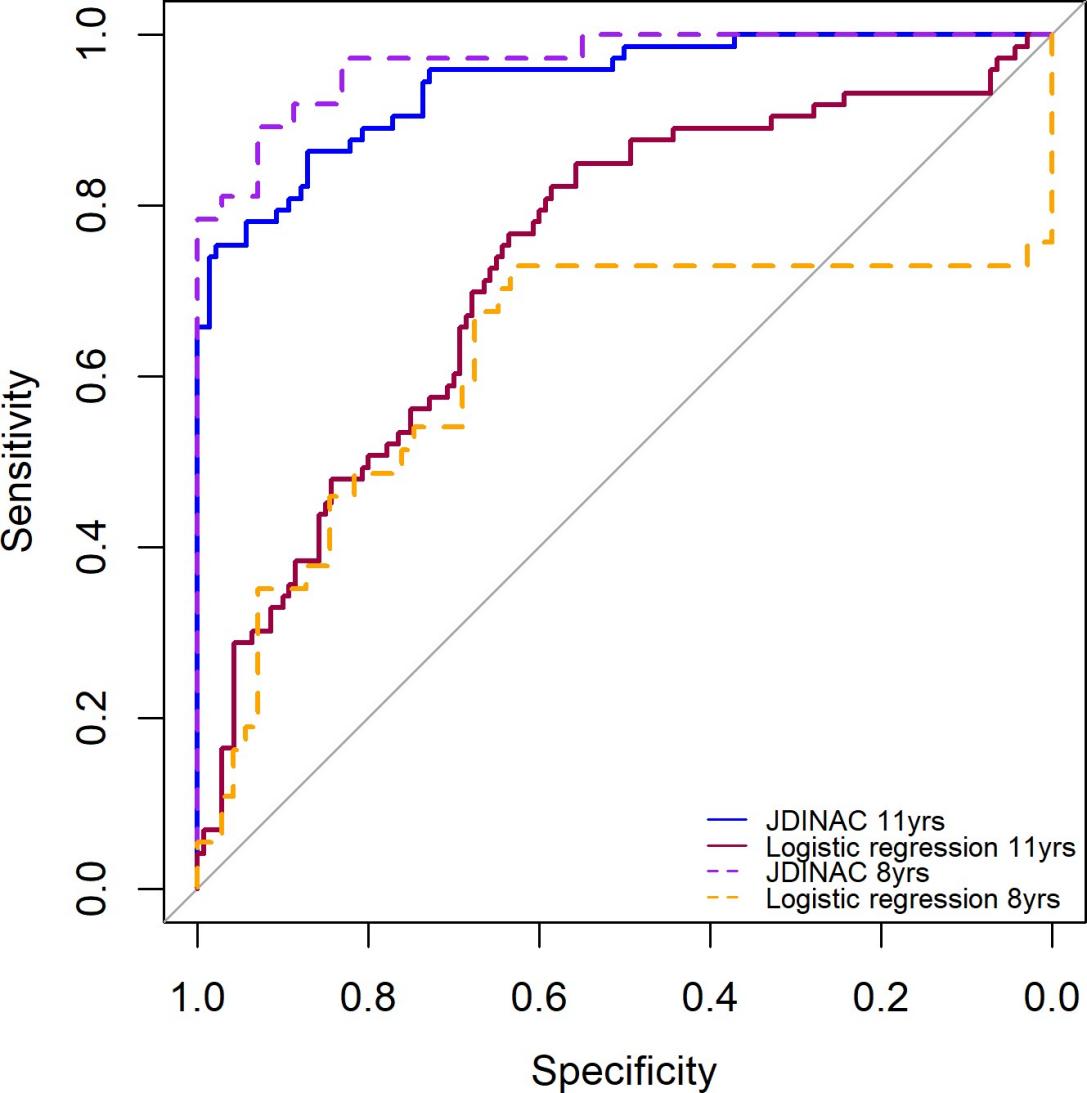
ROC curves for JDINAC and penalised logistic regression. The curves were obtained through the prediction averaging procedure on 50 independent repetitions combined with of 10-fold cross validation. JDINAC, joint density-based nonparametric differential interaction network analysis and classification; ROC, receiver operating characteristic.

Penalised logistic regression with individual components had poor performance, with low sensitivity (0.60) and moderate specificity (0.70). It did not provide an efficient classification rule. In contrast, JDINAC provided a good balance between sensitivity (0.84) and specificity (0.87). Results from 10-fold cross validation in 50 independent repetitions on the whole data set showed that JDINAC with pairwise interaction outperformed penalised logistic regression with individual components, with area under the curve (AUC) equal to 0.94, compared with 0.73 (Fig 5).

These results suggest that the interactions between pairs of sIgE are more informative than the individual components in asthma classification.

**Fig 6:**
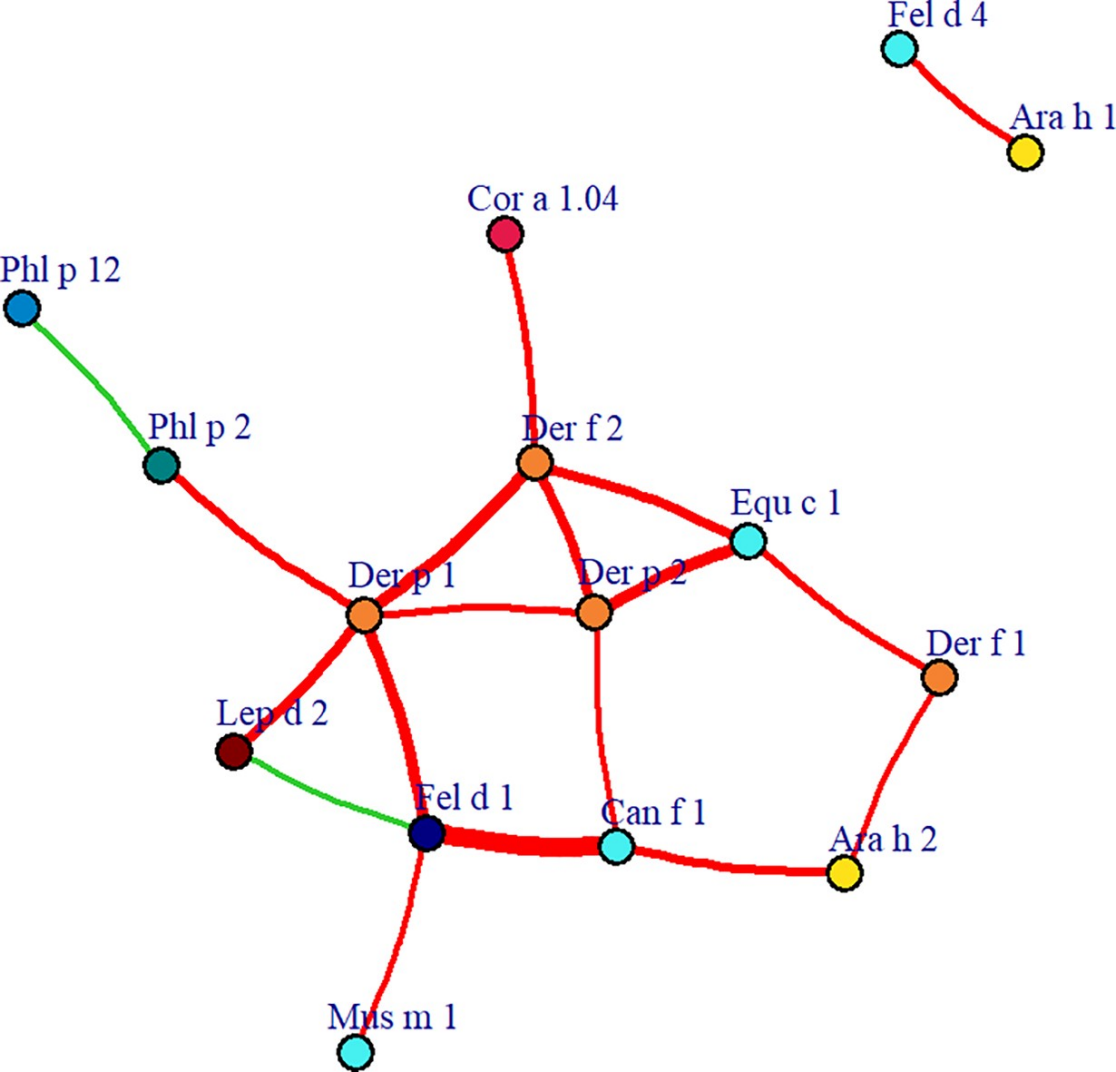
Differential pairwise component-specific IgE interactions in asthma estimated by JDINAC. The presence of an edge presented in the differential network means that the dependency of corresponding pair sIgEs is different between those who have asthma and those who do not have asthma. The edge colour indicates the direction of association. Red: interaction linked to asthma presence; green: interaction linked to reduced risk of asthma. Edge width is proportional to differential weight. Only pairs of sIgEs that were significantly associated to the risk of asthma in 25% of the validation runs were included in the network. IgE, immunoglobulin E; JDINAC, joint density-based nonparametric differential interaction network analysis and classification; sIgE, specific immunoglobulin E.

We then proceeded to infer the differential network of pairwise component-specific IgE interactions that predict asthma by connecting the sIgEs pairs with high differential dependency weights (defined as the number of repetitions in which ${\hat{\beta }}_{ij}\neq 0$). A total of 18 pairs of component-specific IgEs exhibited a significant differential interaction between children who have asthma and children who do not have asthma (Fig 6). The network emphasises multisource connections. HDM and animal components, which were central to the network, showed higher connectivity than other components. The interactions between the grass-related sIgEs (*Phl p 2* and *Phl p 12*) and between *Lep d 2* and *Fel d 1* were linked to a healthy state. In contrast, the remaining pairwise interactions were linked to asthma. The connections between *Fel d 1* and *Can f 1*, *Der p 1* and *Equ c 1*, and *Der f 2* and *Der p 1* had a strong impact on the prediction results because of the higher differential weights.

#### External narrow validation

Of 899 children who attended follow-up at age 8 years, CRD data were obtained for 543 (60.4%). After removing 266 children who had CRD data at age 11 and were hence involved in the previous analyses (S1 Fig), 226 (41.6%) participants were included in the validation set. The filtering procedure resulted in a final sample composed of 108 children who had at least one of the 31 active components >0.30 ISU, of whom 37 (34.2%) had asthma at follow-up at age 8. A flowchart of participants included in the primary analysis and validation is presented in S6 Fig.

**Fig 7:**
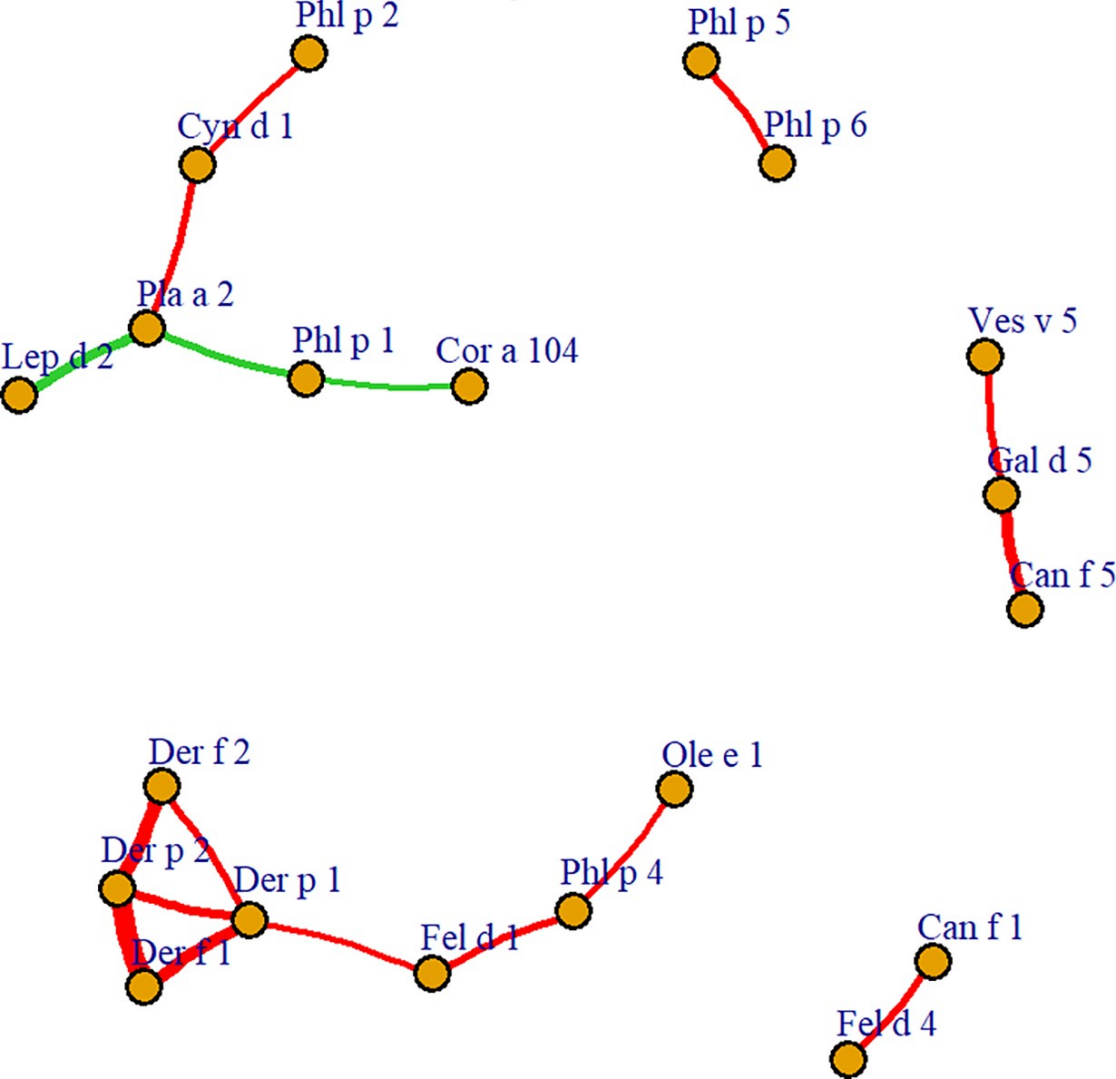
Differential pairwise component-specific IgE interactions in asthma estimated by JDINAC on the 8-year-old children data set. IgE, immunoglobulin E; JDINAC, joint density-based nonparametric differential interaction network analysis and classification; sIgE, specific immunoglobulin E.

JDINAC and penalised logistic regression were run with 10-fold cross validation in 50 independent repetitions. Results were consistent with primary analyses (Table 2) in that penalised logistic regression had low sensitivity (0.46) and high specificity (0.85), whereas JDINAC provided a good balance between sensitivity (0.79) and specificity (0.98). JDINAC had superior performance in classifying asthma, with AUC of 0.97 compared with 0.62 (Fig 5). Most differential pairwise component-specific IgE interactions previously found were confirmed (Fig 7). In particular, pairwise interactions between HDM and animal components had higher differential weights and hence a strong impact on the prediction result, while connectivity between IgE to grass- and tree-related components showed protective pairwise interactions.

## Discussion

### Key findings

Our study suggests that the relationship between allergic sensitisation and asthma is complex and cannot be fully captured or explained by considering sIgE responses to any individual allergenic molecule(s). In contrast to IgE-mediated food allergy, in which sensitisation to a limited number of ‘informative’ allergenic proteins differentiates between true food allergy and asymptomatic sensitisation (such as *Ara h 2* in peanut allergy) [9], we did not identify such ‘informative’ component(s) as a hallmark of an increased risk of asthma. By clustering component-specific IgE responses only (i.e., not the children), we identified seven clusters of component-specific sensitisation, with cluster membership mapped closely to the structural homology of proteins and their biological source. By clustering study participants, we identified four sensitisation clusters that were characterised by unique patterns of sensitisation to allergenic molecules from different component clusters. In this study, the analysis of the relationship between component clusters, sensitisation clusters, and asthma revealed that the key associate of asthma was the interaction between component-specific IgEs, indicating that the important feature of IgE response linked to an increased risk of asthma is not individual IgE to any informative component(s), but the pattern of interactions between component-specific IgEs. Further analyses revealed a differential network of pairwise interactions between a limited number of component-specific IgEs from different component clusters, which predicted asthma with a good balance between sensitivity and specificity. In this study, we found that amongst sensitised children, some of these connectivities were associated with an increased risk of asthma (e.g., between *Fel d 1* and *Can f 1*, *Der p 1* and *Equ c 1*), while others decreased the risk (e.g., between sIgEs to grass components *Phl p 1* and *Phl p 5*).

### Limitations

One of the limitation of our study is that there may be a number of potentially important allergens that are not included on the ISAC chip (e.g., those from fungi), and it is possible that the clustering would provide different solutions if additional components had been available [13]. We acknowledge that our analysis identified only pairwise interactions, and that the relation between asthma and the connectivity structure of sIgE may be more complex. Hence, higher-order interactions will need to be investigated in the future. Furthermore, because of the iterative nature of the JDINAC estimation procedure, we could not estimate the association strength of the differential pairwise interactions. The interpretation is therefore limited to the direction of the association, and further improvements in model design and further validations are needed to fully capitalise on the potential of these findings.

We acknowledge that through our filtering process [19], some potentially important allergens may have been excluded. However, the filtering process was necessary to moderate the effect of measurement errors and noise. Zero-inflated variables can reduce accuracy and usefulness of a cluster analysis, as well as the reliability of the prediction model results. Filtering also increased the confidence of discovering significant association between sIgEs and clinical outcomes of interest. However, we cannot rule out that, despite their rarity, some of the ‘inactive’ components might be associated with asthma and that the inclusion of inactive components might have resulted in different clusters and classification results. We also acknowledge that our findings do not take into account potentially important factors, such as gender and ethnicity, and that they are derived and validated in the same birth cohort (although among different study participants). Therefore, further validations in external populations are needed to ascertain the generalisability of our findings and to evaluate the presence of population-specific characteristics.

### Interpretation

In our previous study using machine learning techniques, we identified three patterns of IgE responses to multiple allergens in the same study population, and each of these patterns was associated with different risk for having asthma [13]. In the current study, we identified seven component clusters that mapped closely to the structural homology of proteins and their biological source (PR-10 proteins, profilins, lipocalins, peanut, grass, trees, and mite clusters). These patterns can be explained by the structural relationships of the allergen components within protein families. The current analysis provided considerably finer granularity compared with our previous analysis, which used Expectation Propagation algorithm implemented in Infer.NET [13]. One possible explanation may be that current methodologies were able to uncover nonlinear relations between the components. Our findings of component clusters are consistent with previous observations that sensitised individual may have detectable IgE to multiple members of the same protein family [36]. For example, one previous study has shown a direct relationship between different representative molecules within three 'panallergen' groups (tropomyosins, profilins, and PR-10s) but little evidence of sensitisation to more than one panallergen [36]. In contrast, our study using a machine learning approach has shown that the PR-10 proteins cluster was central to the network of connectivities and mediated connections between components from other clusters.

Using CRD, several studies have shown that sensitisation to component-specific IgEs is an important risk factor for asthma [37–39]. However, most current guidelines do not recommend assessment of allergic sensitisation as an objective test for asthma diagnosis. This is not surprising, given that in respiratory allergy, the interpretation of SPTs and blood tests that measure specific serum IgE to whole allergen extracts traditionally relies on arbitrary cutoffs (e.g., SPTs > 3 mm, sIgE > 0.35 kUA/L), which have a relatively poor ability to distinguish between benign sensitisations and clinically relevant (‘pathologic’) sensitisation [1, 2]. For example, UK National Institute of Health and Care Excellence (NICE) guidance on the diagnosis of childhood asthma proposes a diagnostic algorithm that incorporates the sequential use of four measures of lung function and inflammation (spirometry, bronchodilator reversibility, fractional exhaled nitric oxide, and peak flow variability, https://www.nice.org.uk/guidance/ng80). We have recently tested the NICE algorithm in a cross-sectional analysis amongst children in our birth cohort aged 13–16 years and found poor agreement between the algorithm and asthma diagnosis; adherence to the algorithm resulted in a substantial number of false positive diagnoses, and the majority of children with asthma were not identified as such by adhering to the proposed algorithm [40]. It is clear that no single test exists for the diagnosis of asthma in children, and using any objective test for diagnosing childhood asthma remains challenging [41]. One important question is whether incorporation of better tests or interpretation algorithms for the assessment of allergic sensitisation would improve diagnostic algorithms for asthma, both in terms of confirming asthma diagnosis and for the assessment of future risk (e.g., of asthma exacerbations or disease persistence). The results of our current study support our notion that ‘allergic sensitisation’ is heterogeneous [4], and provide further evidence that there are several distinct subgroups of sensitisation that differ in their association with asthma. In our previous studies, which used machine learning to investigate patterns of skin test and IgE data to whole extracts of eight major allergens collected at multiple time points throughout childhood, we have shown that some, but not all, classes of sensitisation are associated with asthma presence, progression, and severity [4, 5]. However, these subtypes (clusters/classes) of allergic sensitisation have been identified using statistical inference on large amounts of data collected over long periods [4, 5], and their differentiation at any single cross-sectional point was not possible [42, 43]. Therefore, these observations could not be translated into clinical practice, in which a physician sees a patient at a single time point. It is clear that disaggregation of sensitisation, and knowing which subtype a patient belongs to, may help clinicians predict whether a sensitised patient is likely to have asthma. Our current analysis provides evidence that by using machine learning–based methodologies on CRD data, we can develop better diagnostic algorithms to help practicing physicians differentiate between benign and clinically important allergic sensitisation to help asthma diagnosis [44]. It is of note that our previous studies, which used machine learning but incorporated measures of sensitisation using whole allergen extracts (rather than CRD), were markedly inferior in predicting asthma [12, 45]. Furthermore, compared with our previous studies, in which prediction models correctly classified only one state [12, 45], JDINAC correctly distinguished between children who have asthma and children who do not have asthma.

Another important question is whether similar approaches on CRD data can be used for the assessment of future risk (e.g., of asthma exacerbations) and the prediction of asthma persistence and later-life lung function and chronic obstructive pulmonary disease (COPD) outcomes [6, 7]. In two population-based birth cohorts from the UK and Sweden, we have recently shown IgE reactivity to a limited number of components in preschool identified children at high risk of asthma in adolescence [46]. Persistent asthma at age 16 years in Sweden was predicted by IgE reactivity in early life to four risk molecules (peanut *Ara h 1*, birch *Bet v 1*, cat *Fel d 1*, and grass *Phl p 1*), whilst in the UK, similar association was observed for five allergenic components (dust mite *Der p 1* and *Der f 2*, timothy grass *Phl p 1* and *Phl p 5*, and cat *Fel d 1*) [46]. We have also shown that different longitudinal trajectories of sensitisation to allergenic molecules from timothy grass and HDM during childhood had different associations with subsequent asthma [14]. These data suggest that understanding developmental pathways of IgE responses to multiple allergenic components may help development of prognostic algorithms for asthma. To address this, we recently applied novel machine learning techniques to CRD sensitisation data throughout childhood to describe the architecture of the evolution of IgE responses to >100 allergen components from infancy to adolescence [19]. This analysis has shown that the timing of onset of specific patterns of sensitisation may be a key indicator of the subsequent risk. The above studies show that better resolution of longitudinal patterns of sensitisation to multiple allergenic components may facilitate the development of prognostic algorithms that can be used for the prediction of future risk of asthma. Based on the current results, we propose that the pattern of interactions between component-specific IgEs may provide additional valuable information.

## Conclusion

Our findings suggest that sIgE responses to multiple allergenic proteins are functionally coordinated and co-regulated, and that the patterns of interactions within this complex network may predict clinical phenotypes. In this study, we found that interactions between a limited set of component-specific sIgEs, rather than individual ‘informative’ components, are associated with increased risk of asthma and may provide the basis for designing diagnostic tools. We need to fundamentally rethink the way we interpret data obtained using CRD and move away from the focus on individual component-specific IgEs to a more holistic approach that takes into account the patterns of connectivity between IgEs.

## Supporting information

S1 Appendix Further details on data sources/measurement and definition of outcomes. (DOCX)

S1 Table Characteristics of children IgE data at age 11 years. IgE, immunoglobulin E.
(DOCX)

S2 Table Allergen components to which fewer than 5% of children reacted (inactive components). (DOCX)

S3 Table IgE responses to 44 active components, and differences in the proportion of sensitised children between children who have asthma and children who do not have asthma. IgE, immunoglobulin E.
(DOCX)

S4 Table *χ*^2^test to evaluate the association between clinical outcomes and cluster membership. (DOCX)

S5 Table Relative risk for association between clinical outcomes and cluster membership. (DOCX)

S6 Table Kruskal–Wallis test to evaluate the association between lung function measures and cluster membership. (DOCX)

S1 Fig Flowchart of the analysis steps involved in the study. (TIF)

S2 Fig Distribution of the total number of positive IgE responses to 112 components stratified by asthma status. IgE, immunoglobulin E.
(TIF)

S3 Fig Statistical grouping of allergen components: cluster stability. (TIF)

S4 Fig Patterns of sensitisation among study participants: Cluster stability. (TIF)

S5 Fig Bipartite subnetworks of a subset of sIgE clusters. sIgE, specific immunoglobulin E.
(TIF)

S6 Fig Flowchart of participants in both primary analysis and validation. (TIF)

## Abbreviations

AD: atopic dermatitis
AUC: area under the curve
COPD: chronic obstructive pulmonary disease
CRD: component-resolved diagnostics
HC: hierarchical clustering
HDM: house dust mite
IgE: immunoglobulin E
ISAC: ImmunoCAP Immuno‑Solid phase Allergy Chip
ISU: ISAC Standardised Unit
JDINAC: joint density-based nonparametric differential interaction network analysis and classification
MDS: multidimensional scaling
NICE: UK National Institute of Health and Care Excellence
OR: odds ratio
PAM: partition around medoids
PR: pathogenesis-related
ROC: receiver operating characteristic
sIgE: specific immunoglobulin E
SPT: skin prick test
