# Supplementary material for: Machine learning to identify pairwise interactions between specific IgE antibodies and their association with asthma: A cross-sectional analysis within a population-based birth cohort
Source: PLoS Med. 2018 Nov 13;15(11):e1002691. doi: 10.1371/journal.pmed.1002691 (PMC6233916; doi:10.1371/journal.pmed.1002691)
Supplement: S1 Appendix — (DOCX) [file pmed.1002691.s001.docx]

**S1 Appendix: Further details on data sources/measurement and definition of outcomes.**

***Screening & Recruitment***

All pregnant women were screened for eligibility at antenatal visits (8^th^-10^th^ week of pregnancy) between 1995 and 1997. Of the 1499 couples who met the inclusion criteria (<10 weeks of pregnancy, maternal age >18 years), 288 declined to take part and 27 were lost to follow-up between recruitment and birth of a child. A total of 1184 participants had some evaluable data.

***Follow-up***

Children have been followed prospectively and attended review clinic at age 11 years. We carried out home visits for study participants who could not attend clinic appointments.

***Allergic sensitization:*** Ascertained by skin prick testing (*D pteronyssinus*, cat, dog, grass pollen, moulds, milk, egg, birch and peanut [Bayer, Elkahrt, IN, USA].

***Definition of clinical outcomes***

*Current eczema:* Positive answer to “Has your child had an itchy rash that comes and goes in the last 12 months?”[1]

C*urrent rhinitis:* Positive answer to the question “In the past 12 months, has your child ever had a problem with sneezing, or a runny nose, or a blocked nose when he/she did not have a cold or the flu which was accompanied by itchy-watery eyes?”

*Allergic sensitization:* SPT mean weal diameter 3mm greater than negative control.

1. Asher, M.I., et al., *International Study of Asthma and Allergies in Childhood (ISAAC): rationale and methods.* European Respiratory Journal, 1995. **8**(3): p. 483.
