# Supplementary material for: Machine learning to identify pairwise interactions between specific IgE antibodies and their association with asthma: A cross-sectional analysis within a population-based birth cohort
Source: PLoS Med. 2018 Nov 13;15(11):e1002691. doi: 10.1371/journal.pmed.1002691 (PMC6233916; doi:10.1371/journal.pmed.1002691)
Supplement: S1 Table — IgE, immunoglobulin E. (DOCX) [file pmed.1002691.s002.docx]

**S1 Table. Characteristics of children IgE data at age 11 years**

| Participants’ characteristics | N=461 |
| --- | --- |
|  | **n (%)** |
| Gender (male) | 255 (55.3) |
| Current eczema | 87 (18.9) |
| Current wheeze | 91 (19.7) |
| Allergic Rhinitis | 151 (32.7) |
| Maternal smoking | 59 (12.8) |
| Maternal asthma | 78 (16.9) |
| Paternal asthma | 71 (15.4) |
| Maternal atopy | 256 (55.5) |
| Paternal atopy | 277 (60.1) |
|  | **Median [IQR]** |
| Breastfeeding weeks | 12.00 [1.00 - 28.00] |
| Height | 148.40 [142.90- 153.30] |
| Weight | 40.60 [35.50- 45.75] |
| BMI | 18.31 [16.89-20.38] |
| % Predicted FEV | 98.89 [92.06- 106.36] |
