# Supplementary material for: Machine learning to identify pairwise interactions between specific IgE antibodies and their association with asthma: A cross-sectional analysis within a population-based birth cohort
Source: PLoS Med. 2018 Nov 13;15(11):e1002691. doi: 10.1371/journal.pmed.1002691 (PMC6233916; doi:10.1371/journal.pmed.1002691)
Supplement: S2 Table — (DOCX) [file pmed.1002691.s003.docx]

**S2 Table. Allergen components to which fewer than 5% of children reacted (inactive components)**

| **Inactive components (sIgE <5% positive responses)** | | | | | | | | |
| --- | --- | --- | --- | --- | --- | --- | --- | --- |
| **Act d 1** | **Act d 2** | **Act d 5** | **Act d 8** | **Alt a 6** | **Amb a 1** | **Ana o 2** | **Ani s 1** | **Ani s 3** |
| 2.5% | 3.8% | 0.0% | 1.7% | 1.3% | 0.0% | 0.0% | 1.3% | 4.2% |
| **Api g 1** | **Api m 1** | **Api m 4** | **Ara h 3** | **Ara h 9** | **Art v 1** | **Art v 3** | **Asp f 1** | **Asp f 3** |
| 4.2% | 0.0% | 0.4% | 4.2% | 0.8% | 2.1% | 1.3% | 0.4% | 1.3% |
| **Asp f 6** | **Ber e 1** | **Bet v 4** | **Bla g 1** | **Bla g 2** | **Bla g 5** | **Bla g 7** | **Bos d 4** | **Bos d 5** |
| 2.1% | 0.8% | 3.4% | 1.7% | 0.4% | 0.0% | 3.4% | 0.8% | 0.8% |
| **Bos d 6** | **Bos d 8** | **Bos d lactoferrin** | **Can f 2** | **Can f 3** | **Cla h 8** | **Cor a 8** | **Cor a 9** | **Equ c 3** |
| 1.7% | 0.8% | 0.4% | 3.8% | 3.8% | 0.4% | 0.4% | 0.4% | 2.1% |
| **Fag e 2** | **Fel d 2** | **Gad c 1** | **Gal d 1** | **Gal d 2** | **Gal d 5** | **Gly m 5** | **Gly m 6** | **Hev b 1** |
| 0.0% | 4.6% | 0.4% | 1.3% | 1.3% | 0.0% | 0.8% | 2.1% | 0.8% |
| **Hev b 3** | **Hev b 5** | **Hev b 6.01** | **Jug r 1** | **Jug r 3** | **Ole e 7** | **Ole e 9** | **Par j 2** | **Pen m 1** |
| 0.8% | 0.0% | 1.3% | 1.7% | 1.7% | 1.3% | 2.9% | 0.8% | 2.9% |
| **Pen m 2** | **Pen m 4** | **Phl p 7** | **Pla a 1** | **Pla a 3** | **Pla l 1** | **Pol d 5** | **Pru p 3** | **Sal k 1** |
| 2.1% | 0.8% | 4.6% | 1.7% | 1.3% | 1.7% | 2.1% | 2.5% | 0.0% |
| **Ses I 1** | **Tri a 14** | **Tri a 19** | **Tri a aA_TI** | **Ves v 5** |  |  |  |  |
| 0.8% | 0.0% | 1.3% | 2.1% | 3.4% |  |  |  |  |
