# Supplementary material for: Machine learning to identify pairwise interactions between specific IgE antibodies and their association with asthma: A cross-sectional analysis within a population-based birth cohort
Source: PLoS Med. 2018 Nov 13;15(11):e1002691. doi: 10.1371/journal.pmed.1002691 (PMC6233916; doi:10.1371/journal.pmed.1002691)
Supplement: S3 Table — IgE, immunoglobulin E. (DOCX) [file pmed.1002691.s004.docx]

**S3 Table.** **IgE responses to 44 active components, and differences in the proportion of sensitised children between asthmatic and non-asthmatic children.**

We used Bonferroni correction to adjust for multiple testing.

| Component | No asthma Frequency (%) | Asthma Frequency (%) | p-value | Adjusted p value |  |
| --- | --- | --- | --- | --- | --- |
| *Aln g 1* | 19 (13.6%) | 23 (31.5%) | 0.003 | 0.144 |  |
| *Alt a 1* | 11 (7.9%) | 9 (12.3%) | 0.415 | 1.000 |  |
| *Ara h 1* | 4 (2.9%) | 9 (12.3%) | 0.015 | 0.648 |  |
| *Ara h 2* | 7 (5.0%) | 12 (16.4%) | 0.012 | 0.507 |  |
| *Ara h 6* | 7 (5.0%) | 11 (15.1%) | 0.025 | 1.000 |  |
| *Ara h 8* | 10 (7.1%) | 8 (11.0%) | 0.490 | 1.000 |  |
| *Bet v 1* | 41 (29.3%) | 27 (37.0%) | 0.322 | 1.000 |  |
| *Bet v 2* | 17 (12.1%) | 14 (19.2%) | 0.239 | 1.000 |  |
| *Blo t 5* | 3 (2.1%) | 6 (8.2%) | 0.083 | 1.000 |  |
| *Can f 1* | 11 (7.9%) | 25 (34.2%) | 0.000 | 0.000 | *** |
| *Can f 5* | 10 (7.1%) | 12 (16.4%) | 0.060 | 1.000 |  |
| *Che a 1* | 6 (4.3%) | 10 (13.7%) | 0.028 | 1.000 |  |
| *Cor a 1.01* | 13 (9.3%) | 16 (21.9%) | 0.019 | 0.846 |  |
| *Cor a 1.04* | 24 (17.1%) | 23 (31.5%) | 0.026 | 1.000 |  |
| *Cry j 1* | 7 (5.0%) | 5 (6.8%) | 0.808 | 1.000 |  |
| *Cup a 1* | 14 (10.0%) | 12 (16.4%) | 0.254 | 1.000 |  |
| *Cyn d 1* | 70 (50.0%) | 42 (57.5%) | 0.368 | 1.000 |  |
| *Der f 1* | 49 (35.0%) | 42 (57.5%) | 0.003 | 0.115 |  |
| *Der f 2* | 52 (37.1%) | 46 (63.0%) | 0.001 | 0.025 | * |
| *Der p 1* | 53 (37.9%) | 43 (58.9%) | 0.005 | 0.236 |  |
| *Der p 10* | 0 (0.0%) | 7 (9.6%) | 0.001 | 0.039 | * |
| *Der p 2* | 50 (35.7%) | 44 (60.3%) | 0.001 | 0.046 | * |
| *Equ c 1* | 4 (2.9%) | 15 (20.5%) | 0.000 | 0.002 | * |
| *Fel d 1* | 44 (31.4%) | 33 (45.2%) | 0.066 | 1.000 |  |
| *Fel d 4* | 6 (4.3%) | 14 (19.2%) | 0.001 | 0.044 | * |
| *Gal d 3* | 4 (2.9%) | 9 (12.3%) | 0.015 | 0.648 |  |
| *Gly m 4* | 6 (4.3%) | 8 (11.0%) | 0.115 | 1.000 |  |
| *Hev b 8* | 22 (15.7%) | 15 (20.5%) | 0.488 | 1.000 |  |
| *Jug r 2* | 8 (5.7%) | 8 (11.0%) | 0.269 | 1.000 |  |
| *Lep d 2* | 16 (11.4%) | 19 (26.0%) | 0.011 | 0.496 |  |
| *Mal d 1* | 17 (12.1%) | 21 (28.8%) | 0.005 | 0.212 |  |
| *Mer a 1* | 22 (15.7%) | 15 (20.5%) | 0.488 | 1.000 |  |
| *Mus m 1* | 2 (1.4%) | 9 (12.3%) | 0.002 | 0.089 |  |
| *Mux f 3* | 15 (10.7%) | 11 (15.1%) | 0.483 | 1.000 |  |
| *Ole e 1* | 35 (25.0%) | 18 (24.7%) | 1.000 | 1.000 |  |
| *Phl p 1* | 95 (67.9%) | 52 (71.2%) | 0.727 | 1.000 |  |
| *Phl p 11* | 27 (19.3%) | 18 (24.7%) | 0.463 | 1.000 |  |
| *Phl p 12* | 15 (10.7%) | 9 (12.3%) | 0.900 | 1.000 |  |
| *Phl p 2* | 54 (38.6%) | 28 (38.4%) | 1.000 | 1.000 |  |
| *Phl p 4* | 69 (49.3%) | 35 (47.9%) | 0.967 | 1.000 |  |
| *Phl p 5* | 81 (57.9%) | 42 (57.5%) | 1.000 | 1.000 |  |
| *Phl p 6* | 45 (32.1%) | 28 (38.4%) | 0.450 | 1.000 |  |
| *Pla a 2* | 8 (5.7%) | 7 (9.6%) | 0.443 | 1.000 |  |
| *Pru p 1* | 15 (10.7%) | 19 (26.0%) | 0.007 | 0.306 |  |
