# Supplementary material for: Machine learning to identify pairwise interactions between specific IgE antibodies and their association with asthma: A cross-sectional analysis within a population-based birth cohort
Source: PLoS Med. 2018 Nov 13;15(11):e1002691. doi: 10.1371/journal.pmed.1002691 (PMC6233916; doi:10.1371/journal.pmed.1002691)
Supplement: S4 Table — (DOCX) [file pmed.1002691.s005.docx]

**S4 Table. χ^2^ test to evaluate association between clinical outcomes and cluster membership.**

|  |  | Predominantly grass and tree sensitisation | Predominantly HDM sensitisation | Multiple sensitization | Lower-grade sensitisation | p-value |
| --- | --- | --- | --- | --- | --- | --- |
| N= |  | **55** | **47** | **59** | **52** |  |
| Asthma | No | 39 (27.9%) | 26 (18.6%) | 31(22.1%) | 44 (31.4%) | 0.001 |
|  | Yes | 16 (21.9%) | 21 (28.8%) | 28 (38.4%) | 8 (11.0%) |  |
| Wheeze | No | 41 (28.7%) | 22 (15.4%) | 35 (24.5%) | 45 (31.5%) | 0.000 |
|  | Yes | 14 (20.0%) | 25 (35.7%) | 24 (34.3%) | 7 (10.0%) |  |
| Eczema | No | 41 (26.1%) | 36 (22.9%) | 39 (24.8%) | 41 (26.1%) | 0.482 |
|  | Yes | 14 (25.9%) | 10 (18.5%) | 19 (35.2%) | 11 (20.4%) |  |
| Rhinitis | No | 16 (15.7%) | 30 (29.4%) | 18 (17.6%) | 38 (37.3%) | 0.000 |
|  | Yes | 39 (35.1%) | 17 (15.3%) | 41 (36.9%) | 14 (26.9%) |  |
