# Supplementary material for: Machine learning to identify pairwise interactions between specific IgE antibodies and their association with asthma: A cross-sectional analysis within a population-based birth cohort
Source: PLoS Med. 2018 Nov 13;15(11):e1002691. doi: 10.1371/journal.pmed.1002691 (PMC6233916; doi:10.1371/journal.pmed.1002691)
Supplement: S5 Table — (DOCX) [file pmed.1002691.s006.docx]

**S5 Table. Odds ratios for association between clinical outcomes and cluster membership.**

Odds ratios were computed through unconditional maximum likelihood and confidence intervals were estimated using a normal approximation. Computations are performed through the R package *epitools*

|  | Asthma | Wheeze | Eczema | Rhinitis |
| --- | --- | --- | --- | --- |
|  | OR [95% CI]  p-value | OR [95% CI]  p-value | OR [95% CI]  p-value | OR [95% CI]  p-value |
| Lower-grade sensitisation | 1.00 | 1.00 | 1.00 | 1.00 |
| Predominantly  grass and tree sensitisation | 2.25 [0.87-5.84] p=0.108 | 2.19 [0.81-5.97] p=0.147 | 1.27 [0.52-3.13] p=0.652 | 6.62 [2.84-15.40] p<0.001 |
| Predominantly  HDM sensitisation | 4.44 [1.72-11.46] p=0.002 | 7.31 [2.74-19.48] p<0.001 | 1.04 [0.39-2.72] p=1.000 | 1.54 [0.65-3.61] p=0.386 |
| Multiple sensitization | 4.97 [1.99-12.34] p<0.001 | 4.41 [1.70-11.41] p<0.001 | 1.82 [0.77-4.30] p=0.202 | 6.18 [2.71-14.12] p<0.001 |
