# Supplementary material for: Machine learning to identify pairwise interactions between specific IgE antibodies and their association with asthma: A cross-sectional analysis within a population-based birth cohort
Source: PLoS Med. 2018 Nov 13;15(11):e1002691. doi: 10.1371/journal.pmed.1002691 (PMC6233916; doi:10.1371/journal.pmed.1002691)
Supplement: S6 Table — (DOCX) [file pmed.1002691.s007.docx]

**S6 Table. Kruskal-Wallis test to evaluate association between lung function measures and cluster membership.**

Results are reported ad median and IQR.

|  | **Predominantly grass and tree sensitisation** | **Predominantly HDM sensitisation** | **Multiple sensitization** | **Lower-grade sensitisation** | **p-value** |
| --- | --- | --- | --- | --- | --- |
| FEV1 % predicted | 100.72  [92.43; 105.49] | 100.40  [92.98; 105.81] | 96.04  [89.87; 104.79] | 99.1 0  [91.16; 108.05] | 0.715 |
| FVC | 2.64  [2.50; 2.85] | 2.71  [2.38; 3.10] | 2.62  [2.29; 2.93] | 2.63  [2.36; 3.03] | 0.463 |
| FEV1/FVC ratio | 0.86  [0.83; 0.90] | 0.86  [0.82; 0.92] | 0.86  [0.80; 0.90] | 0.86  [0.82; 0.91] | 0.714 |
