# Supplementary figures and images for: Machine learning to identify pairwise interactions between specific IgE antibodies and their association with asthma: A cross-sectional analysis within a population-based birth cohort
Source: PLoS Med. 2018 Nov 13;15(11):e1002691. doi: 10.1371/journal.pmed.1002691 (PMC6233916; doi:10.1371/journal.pmed.1002691)

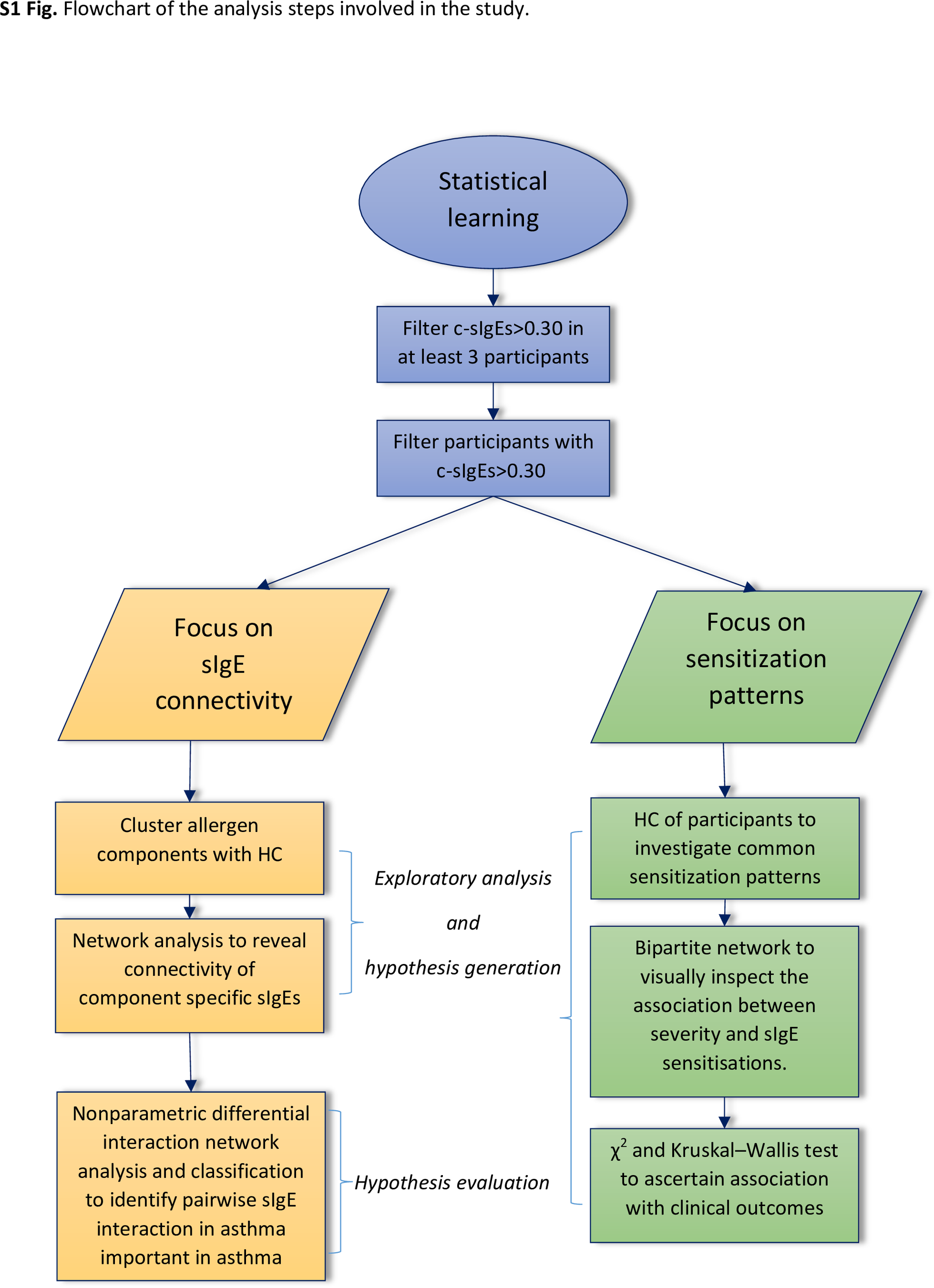

Supplement: S1 Fig — (TIF) [file pmed.1002691.s008.tif]

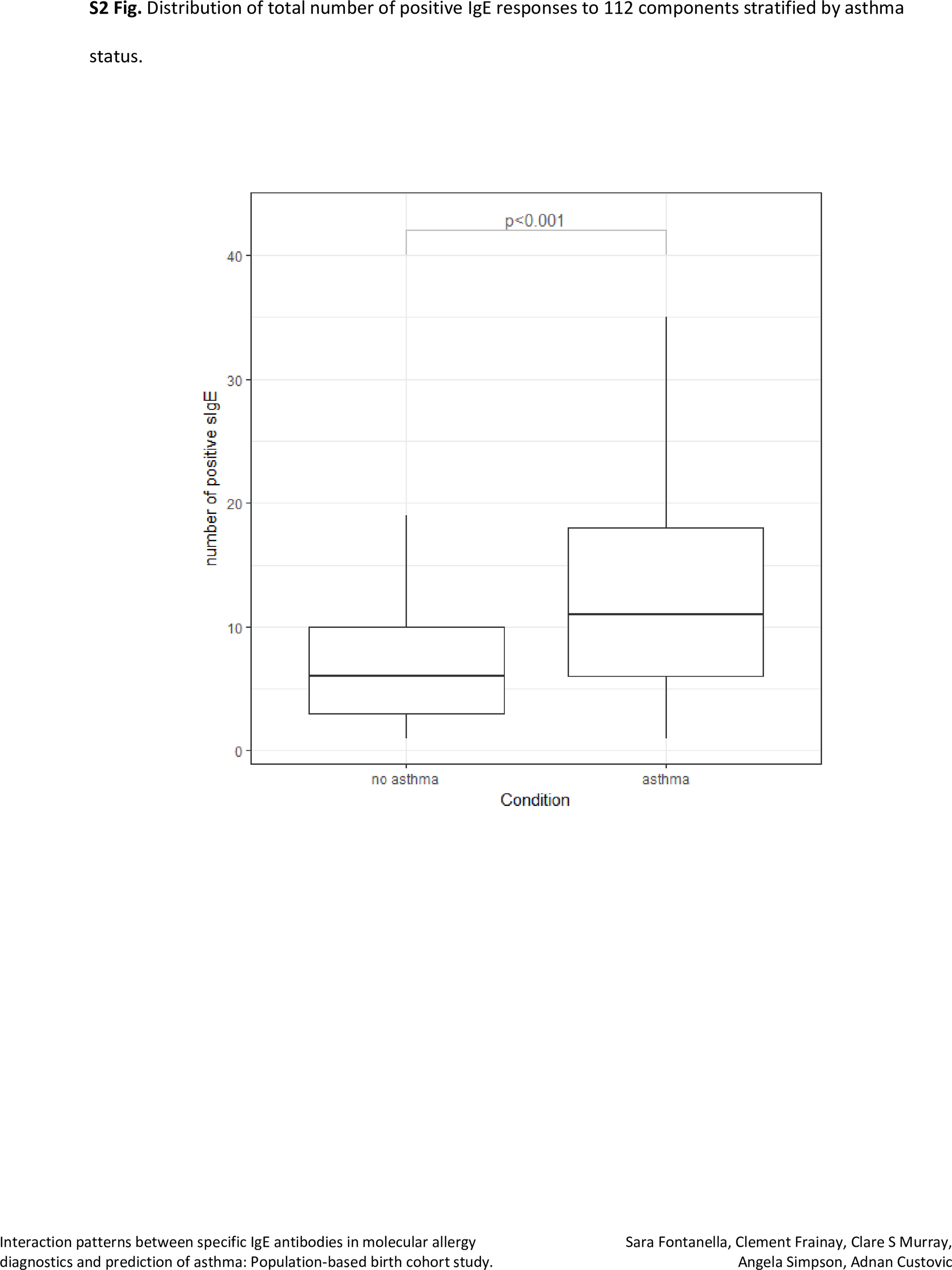

Supplement: S2 Fig — IgE, immunoglobulin E. (TIF) [file pmed.1002691.s009.tif]

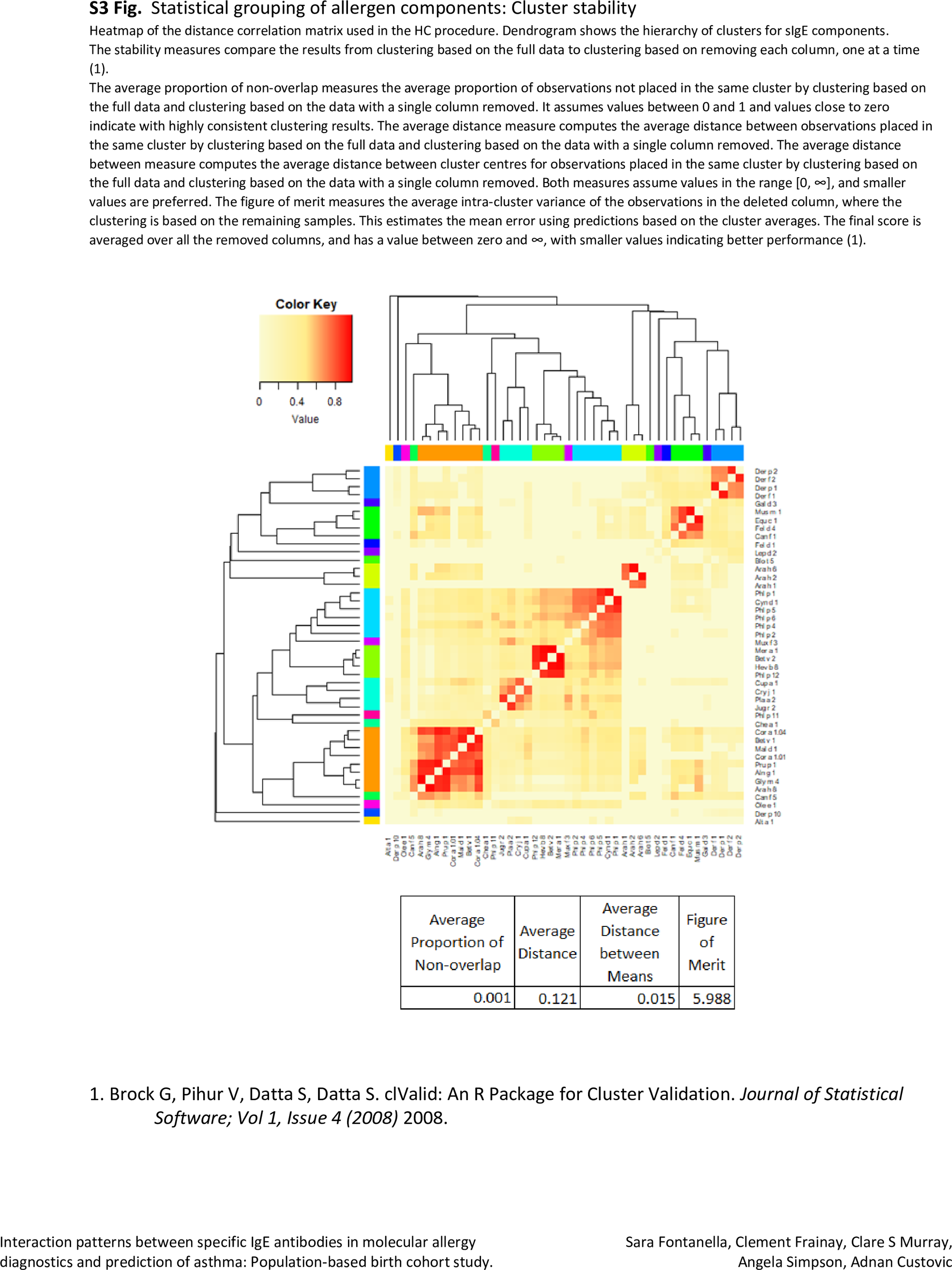

Supplement: S3 Fig — (TIF) [file pmed.1002691.s010.tif]

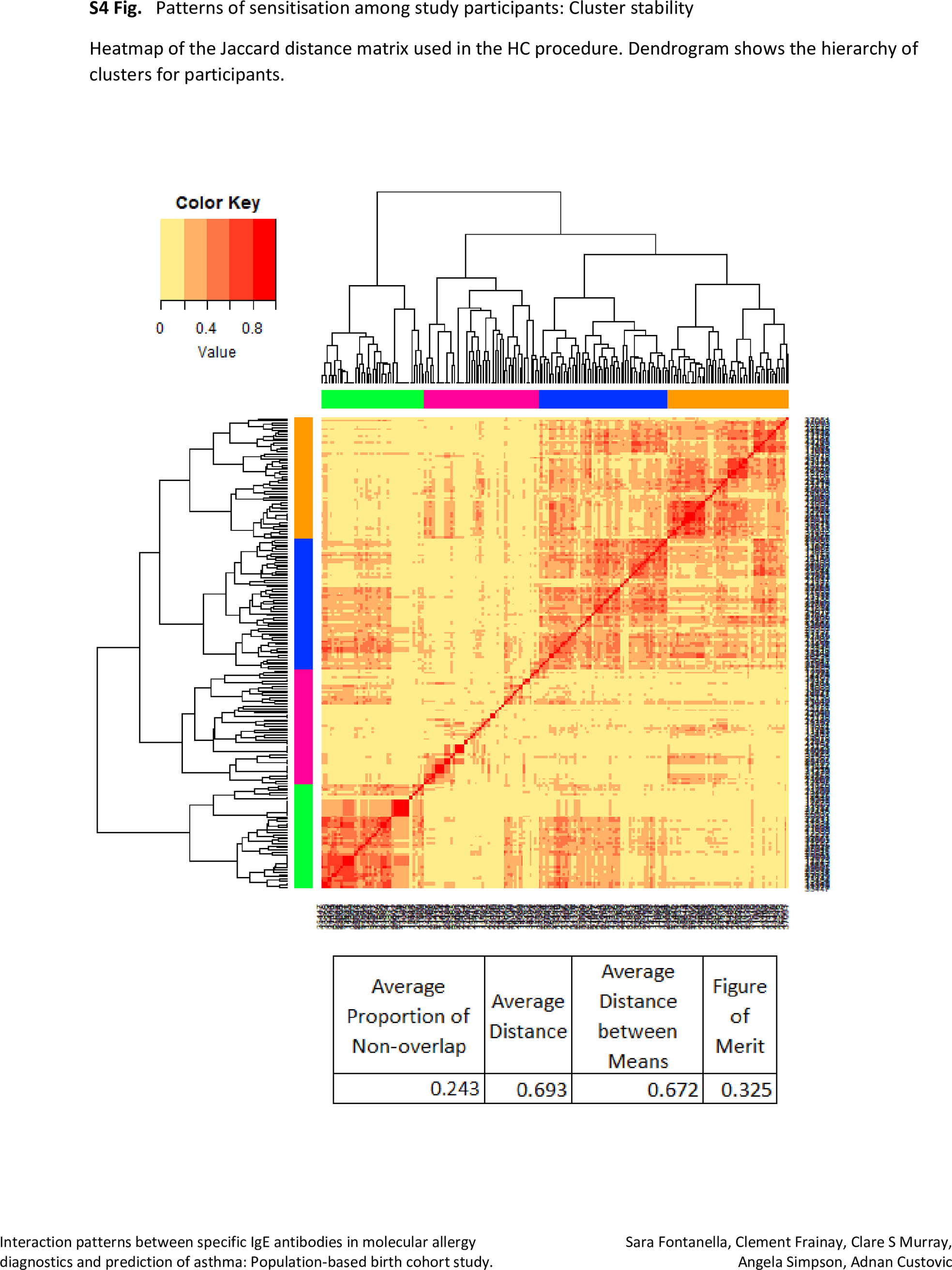

Supplement: S4 Fig — (TIF) [file pmed.1002691.s011.tif]

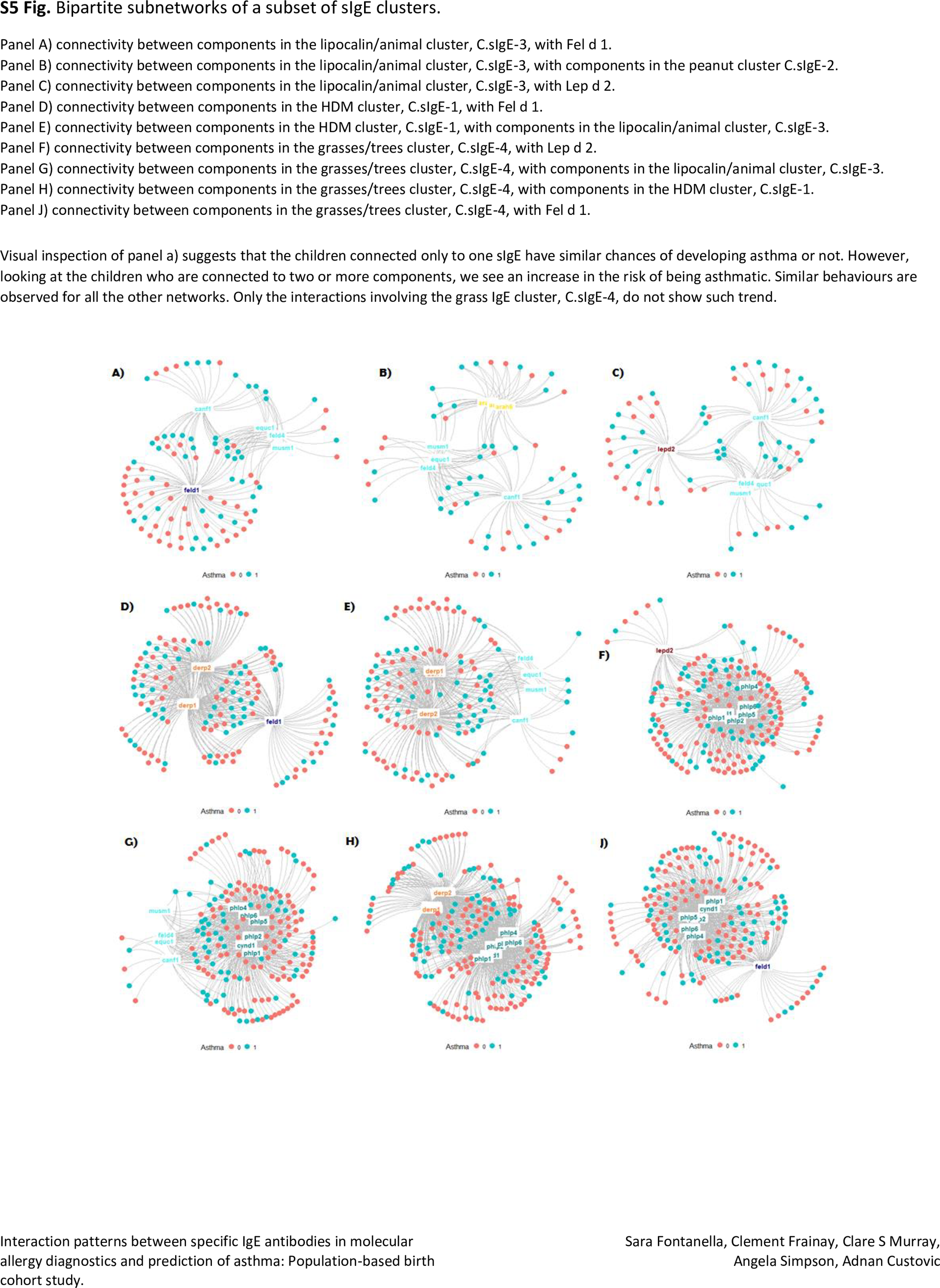

Supplement: S5 Fig — sIgE, specific immunoglobulin E. (TIF) [file pmed.1002691.s012.tif]

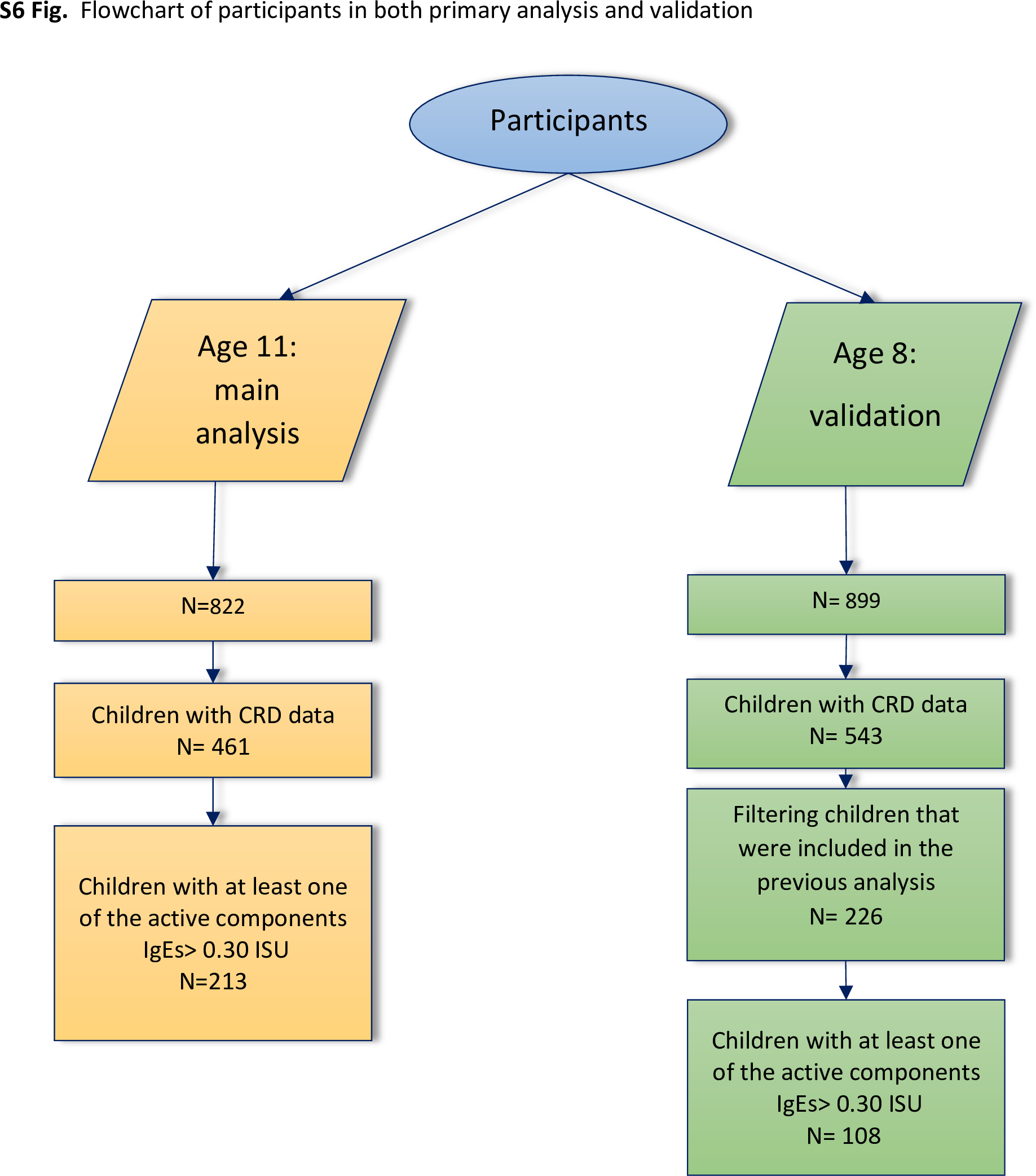

Supplement: S6 Fig — (TIF) [file pmed.1002691.s013.tif]
